# Outer Membrane Vesicles Prime and Activate Macrophage Inflammasomes and Cytokine Secretion *In Vitro* and *In Vivo*

**DOI:** 10.3389/fimmu.2017.01017

**Published:** 2017-08-25

**Authors:** Jessica D. Cecil, Neil M. O’Brien-Simpson, Jason C. Lenzo, James A. Holden, William Singleton, Alexis Perez-Gonzalez, Ashley Mansell, Eric C. Reynolds

**Affiliations:** ^1^Oral Health CRC, Melbourne Dental School, Bio21 Institute, The University of Melbourne, Melbourne, VIC, Australia; ^2^Department of Molecular and Translational Science, Monash University, Clayton, VIC, Australia

**Keywords:** outer membrane vesicles, macrophages, inflammasomes, *Porphyromonas gingivalis*, *Treponema denticola*, *Tannerella forsythia*, periodontitis

## Abstract

Outer membrane vesicles (OMVs) are proteoliposomes blebbed from the surface of Gram-negative bacteria. Chronic periodontitis is associated with an increase in subgingival plaque of Gram-negative bacteria, *Porphyromonas gingivalis, Treponema denticola*, and *Tannerella forsythia*. In this study, we investigated the immune-modulatory effects of *P. gingivalis, T. denticola*, and *T. forsythia* OMVs on monocytes and differentiated macrophages. All of the bacterial OMVs were phagocytosed by monocytes, M(naïve) and M(IFNγ) macrophages in a dose-dependent manner. They also induced NF-κB activation and increased TNFα, IL-8, and IL-1β cytokine secretion. *P. gingivalis* OMVs were also found to induce anti-inflammatory IL-10 secretion. Although unprimed monocytes and macrophages were resistant to OMV-induced cell death, lipopolysaccharide or OMV priming resulted in a significantly reduced cell viability. *P. gingivalis, T. denticola*, and *T. forsythia* OMVs all activated inflammasome complexes, as monitored by IL-1β secretion and ASC speck formation. ASC was critical for OMV-induced inflammasome formation, while AIM2−/− and Caspase-1−/− cells had significantly reduced inflammasome formation and NLRP3−/− cells exhibited a slight reduction. OMVs were also found to provide both priming and activation of the inflammasome complex. High-resolution microscopy and flow cytometry showed that *P. gingivalis* OMVs primed and activated macrophage inflammasomes *in vivo* with 80% of macrophages exhibiting inflammasome complex formation. In conclusion, periodontal pathogen OMVs were found to have significant immunomodulatory effects upon monocytes and macrophages and should therefore influence pro-inflammatory host responses associated with disease.

## Introduction

Chronic periodontitis is an inflammatory disease of the periodontal tissues associated with an increase in Gram-negative bacteria within the subgingival plaque biofilm; in particular, proportional increases in the pathogenic species *Porphyromonas gingivalis, Treponema denticola*, and *Tannerella forsythia* (1–3). During disease progression various bacterial virulence factors, including outer membrane vesicles (OMVs), are released from the subgingival plaque into the subjacent connective tissue where they induce a pro-inflammatory host response (4). Periodontal pathogen OMVs are closed proteoliposomes composed of lipopolysaccharide, lipoproteins, nucleic acids (DNA and RNA), peptidoglycan, porins, and receptors (5–9), which are known to disrupt tight junctions in epithelial monolayers, induce neutrophil and macrophage recruitment, and stimulate strong pro-inflammatory cytokine responses from various host cells (10–12). While inflammation is an important component of the host defense, persistent and dysregulated inflammation provides a nutritionally favorable environment for oral pathogenic bacteria adhered to the tooth root in a periodontal pocket and is largely responsible for the tissue and bone destruction that characterizes periodontitis (13).

Monocytes and macrophages are known to shape the host immune response to bacterial infection through phagocytosis, antigen presentation, and cytokine production. Gingival tissue biopsies from periodontitis patients have shown elevated numbers of macrophages and higher concentrations of nitric oxide synthase and pro-inflammatory cytokines IL-1β, TNFα, IL-8, IL-6, and MIP-1α, which serve to promote inflammation and recruit additional immune cells to the site of infection (14–16). IL-1 family cytokines are significant contributors to inflammation and bone loss during chronic periodontitis and have been correlated with the severity of disease (17, 18). The maturation and secretion of IL-1β is mediated by powerful multiprotein complexes termed inflammasomes, which are found in the cytosol of myeloid cells (19). Inflammasome-induced IL-1β secretion requires two signaling events, an initial “cell priming” through NF-κB to mediate synthesis of pro-IL-1β and a second “triggering” event induced by cell surface or cytosolic receptor recognition of pathogen- or damage-associated molecular patterns (PAMPs/DAMPs) that initiate oligomerization of inflammasome components to form an enzymatic complex that results in the proteolytic maturation and secretion of IL-1β (20). Intriguingly, bacterial OMVs are known to bind to mammalian cells and through a number of mechanisms be rapidly internalized, thus OMVs would deliver PAMPs to both cell surface and cytosolic receptors (21) Several classes of inflammasome exist, including the NLR subsets NLRP1, NLRP3, and NLRC4, of which NLRP3 is the best studied. NLRP3 formation is known to be triggered by a wide range of external and internal stimuli, which prime and activate the inflammasome through signal transduction pathways (22, 23). Direct cytosolic contact with bacterial PAMPs or other stimuli is not necessary to activate the NLRP3 inflammasome (22). The alternative AIM2 inflammasome is stimulated by cytosolic double-stranded DNA, which may be of viral or bacterial origin or resulting from disruption of the nuclear envelope (24). Inflammasome activation also triggers a form of inflammatory cell death, termed pyroptosis, which promotes the rapid release of cytosolic contents (including IL-1β) primarily due to Caspase-1-induced pores in the cell membrane (25), although other caspases are also known to perform this role (26). Gasdermin-D has recently been identified as a major pore-forming protein (27, 28) and can be cleaved by Caspases 1, 4, 5, and 11 to mediate pyroptotic cell death (29). Pyroptosis is an antimicrobial response that not only eliminates intracellular niches for pathogens but can also cause tissue injury, accelerate bacterial dissemination, and inhibit bacterial clearance from tissues (30). Recently, inflammasome components Caspase-1, NLRP3, and AIM2 have been shown to be upregulated in the gingival tissue of periodontitis patients, suggesting that macrophage inflammasome activation may play a significant role in periodontal immune responses (31).

Circulating blood monocytes are differentiated into phenotypically diverse macrophage classes when recruited into periodontal tissues by the early inflammatory response (32). The classic inflammatory M[IFNγ + lipopolysaccharide (LPS)] macrophage, formerly known as M1, is differentiated by early IFNγ exposure followed by TLR ligation, while anti-inflammatory M(IL-4) macrophages, formerly known as M2, are differentiated by IL-4 or IL-13 cytokine exposure (32, 33). This well adapted flexibility allows macrophages to promote, control, or resolve inflammation as required in host tissues. We have shown that M(IFNγ + LPS) macrophages are the dominant infiltrating macrophage in mouse periodontitis models followed by monocytes and undifferentiated M(naïve) class macrophages and are crucial for disease progression (34). Despite their pathogenic potential, few studies have explored the effects of periodontal pathogen OMVs on monocytes and macrophages. *P. gingivalis* OMVs are reported to induce nitric oxide production (35) and foam cell formation in macrophages (36). Yet gingipains on *P. gingivalis* OMVs are also reported to promote immune evasion by the proteolytic degradation of membrane-bound LPS receptor CD14 on human macrophages (37). *T. forsythia* OMVs are known to induce pro-inflammatory cytokine release from macrophages and periodontal fibroblast cell lines (38). While no study to date has explored the effects of *T. denticola* OMVs on macrophages or monocytes, outer membrane lipoproteins, and lipooligosaccharide, known to be present on *T. denticola* OMVs (39), have been shown to stimulate nitric oxide production and strong pro-inflammatory cytokine secretion (TNFα and IL-1β) in murine macrophages (40). To facilitate the development of new therapies for periodontal disease it is vital to understand how inflammation is initiated, controlled, and resolved by immune-modulatory cells in response to bacterial products. The aim of this study was to investigate the interactions between monocytes/macrophages and OMVs derived from oral pathogens.

## Materials and Methods

### Study Design

The objective of this study was to observe the immunomodulatory effects of periodontal OMVs upon THP-1 cells (*in vitro*) and peritoneal macrophages extracted from C57BL/6 J mice (*ex vivo* and *in vivo*). To assess THP-1 monocyte/macrophage interactions we developed *in vitro* OMV binding, phagocytosis, cytokine secretion, and inflammasome activation assays. All *in vitro* experiments were performed at least three times with triplicate samples, data collection was stopped when comparable results were found throughout three experiments.

To evaluate the inflammatory potential of OMVs *in vivo* we compared the stimulation of C57BL/6 J mice treated with intraperitoneal injections of phosphate-buffered saline (PBS), *Escherichia coli* LPS, or *P. gingivalis* OMVs. The sample size was selected on the basis of previous data to allow reliable detection of OMV-induced inflammasome formation. Mice were randomized for inclusion in each of the treatment arms. All mice were included for analysis. Investigators were aware of the allocation sequence, and data were collected and processed in groups, PBS-treated mice then LPS-treated mice, and OMV-treated mice. Inflammasome activation was determined using IL-1β secretion, ASC localization by flow cytometry and high resolution microscopy.

#### Bacterial Cultures and Growth Conditions

All bacterial strains were obtained from the Melbourne Dental School culture collection. *P. gingivalis* W50 was maintained on horse blood agar, comprised of 4% w/v Blood Agar Base No. 2 (Thermo Fisher Scientific, SA, Australia) supplemented with 10% v/v defibrinated horse blood (Equicell, Australia) and 1% w/v menadione (Sigma-Aldrich, NSW, Australia) in a MK3 Anaerobic Workstation (Don Whitley Scientific Limited, NSW, Australia) at 37°C with a gas composition of 5% v/v H_2_, 10% v/v CO_2_ in N_2_ (BOC Gases Australia, NSW, Australia). *P. gingivalis* W50 was cultured in volumes of 800 mL in 3.7% w/v Bacto Brain Heart Infusion media broth (BD, NSW, Australia) supplemented with 0.5% w/v hemin (Merck Millipore, VIC, Australia) and 0.1% w/v cysteine (Sigma-Aldrich, NSW, Australia). Bacteria were grown to late exponential phase at 37°C under anaerobic conditions to an optical density of 1.0 at 600 nm.

*Treponema denticola* ATCC 35405 was cultured in volumes of 400 mL of Oral Bacteria Growth Media comprised of 1.25% w/v Bacto Brain Heart Infusion media broth, 1% w/v tryptic soy broth (BD, NSW, Australia), 0.75% w/v yeast extract (Thermo Fisher Scientific, SA, Australia), 0.2% w/v sodium chloride (VWR International, QLD, Australia), 0.2% w/v ascorbic acid (Sigma-Aldrich, NSW, Australia), 0.2% w/v d-glucose (Chem Supply, SA, Australia), 0.1% w/v pyruvic acid (Sigma-Aldrich, NSW, Australia), 0.05% w/v sodium thioglycolate (Sigma-Aldrich, NSW, Australia), and 0.025% w/v asparagines (Sigma-Aldrich, NSW, Australia) supplemented with 0.2% w/v sodium bicarbonate (Chem Supply, SA, Australia), 0.2% g ammonium sulfate (Chem Supply, SA, Australia), 0.1% w/v cysteine, 0.6% w/v thiamine pyrophosphate (Sigma-Aldrich, NSW, Australia), 0.5% w/v hemin, 0.05% w/v menadione, 2.5% v/v heat-inactivated Rabbit Serum (Sigma-Aldrich, NSW, Australia) filtered using a Vivaspin 10 kDa MWCO (GE HealthCare Life Sciences, NSW, Australia) at 8,000 × *g* for 1 h at 4°C, and 0.0025% v/v volatile fatty acid mix containing 0.5% v/v isobutyric acid, 0.5% v/v dl-1-methylbutyric acid, 0.5% v/v isovaleric acid, and 0.5% v/v valeric acid in 0.1 M potassium hydroxide.

*Tannerella forsythia* ATCC 43037 was cultured in volumes of 400 mL in tryptic soy broth with yeast extract and vitamin K (TSBYK), comprised of 1.85% w/v Bacto Brain Heart Infusion media broth, 1.5% w/v tryptic soy broth, and 1.0% w/v yeast extract supplemented with 0.1% w/v cysteine, 0.1% *N*-acetylmuramic acid (Sigma-Aldrich, NSW, Australia), 0.5% w/v hemin, 0.05% menadione, and 5% v/v heat-inactivated fetal calf serum (HI-FCS) filtered using a Vivaspin 10 kDa MWCO (GE HealthCare Life Sciences, NSW, Australia) at 8,000 × *g* for 1 h at 4°C.

#### OMV Preparation and Enumeration

##### Isolation and Enrichment of Highly Purified OMVs

Highly purified OMVs were isolated and enriched using ultracentrifugation, tangential flow filtration, and density gradient separation as previously described (39). Briefly, bacteria were grown to late exponential phase and removed from culture supernatant by centrifugation. The collected supernatant was filtered (0.22 µm) and then concentrated through a 100-kDa filter using tangential flow filtration. The collected concentrate was centrifuged at 100,000 × *g* for 2 h at 4°C to yield a crude OMV preparation. The crude OMVs were then separated from membrane fragments and other contaminates by a discontinuous OptiPrep™ (Sigma-Aldrich, NSW, Australia) gradient at 150,000 × *g* for 16 or 48 h. Gradient fractions containing the purified OMVs were pooled and washed with 0.01 M PBS (Sigma-Aldrich, NSW, Australia) and stored at 4°C for short-term storage (<14 days) and −80°C for long-term storage (>14 days).

##### Enumerating OMVs

Purified OMVs were counted using PKH-26 Red Fluorescent Cell Linker (Sigma-Aldrich, NSW, Australia) and an Apogee A50-Micro Flow Cytometer calibrated with Apogee Flow Systems Calibration Beads (1.6 µm for Red Laser, 1,030 eV/μL) as previously described (39). The Apogee A50-Micro Flow Cytometer was kindly provided by Prof Frank Caruso (University of Melbourne, Australia).

#### OMV to Cell Binding Assays

The human monocyte cell line THP-1 was cultured in complete Dulbecco’s Modified Eagle’s Medium (DMEM, 10% v/v fetal calf serum, 3.5% v/v glucose, 1% v/v Pen/Strep, 1% v/v l-Glut) in a 5% CO_2_ incubator at 37°C. Cells were used in their native monocyte cell form or differentiated into M(naïve) and M(IFNγ) macrophage-like cells by treatment with 5 nM of phorbol 12-myristate 13-acetate (PMA) for 48 h [M(naïve)] and an additional 24 h treatment with 100 ng/mL of human IFNγ [M(IFNγ)]. Cells were seeded into 96-well flat-bottom tissue culture plates at a volume of 200 µL per well (1 × 10^5^ cells per well). THP-1 cells were incubated with *P. gingivalis, T. denticola*, and *T. forsythia* OMVs labeled with lipid intercalating dye PKH-26 according to the manufacturer’s instructions. Vesicles were added at OMV to cell ratios of 10:1, 50:1, and 100:1. Following incubation at 37°C for 60 min in a 5% CO_2_ incubator, cell media were removed, the cells were removed by trypsin EDTA solution (Sigma-Aldrich, NSW, Australia), washed and resuspended in PBS, and the level of binding determined using flow cytometry methods previously described (41).

#### OMV Phagocytosis Assays

THP-1 cells were seeded into 96-well flat-bottom tissue culture plates at a volume of 200 µL per well (1 × 10^5^ cell per well). THP-1 cells were incubated with *P. gingivalis, T. denticola*, and *T. forsythia* OMVs labeled with pH-sensitive dye pHrodo Red, succinimidyl ester (Life Technologies, Australia) according to the manufacturer’s instructions. Vesicles were added at OMV to cell ratios of 10:1, 50:1, and 100:1. Following incubation at 37°C for 60 min in a 5% CO_2_ incubator, cell medium was removed, the cells were released by trypsin EDTA solution (Sigma-Aldrich, NSW, Australia), washed and resuspended in PBS, and the level of phagocytosis determined using flow cytometry as above.

#### NF-κB Activation Assays

THP-Blue cells (Invivogen, USA) are characterized by the stable integration of an NF-κB-inducible SEAP reporter gene that induces the secretion of alkaline phosphatase following cell stimulation by pattern recognition receptor (PRR) agonists. THP-Blue cells were grown at 37°C in an anaerobic chamber in complete DMEM (10% v/v fetal calf serum, 3.5% v/v glucose, 1% v/v Pen/Strep, and 1% v/v l-Glut). Cells were removed from culture flasks (Corning, VIC, Australia) by gentle tapping and counted using a Z1 Coulter Particle Counter (Beckman Coulter, NSW, Australia). A flat bottomed 96-well plate (Interpath Services, VIC, Australia) was seeded with 2 × 10^4^ cells per well in 200 µL of complete DMEM, differentiated into M(naïve) and M(IFNγ) macrophages as above, and incubated for 24 h at 37°C under anaerobic conditions. Supernatant was aspirated and 200 µL of fresh, warmed DMEM added. THP-Blue cells were challenged with 20 µL of OMVs in PBS starting at 1.0 × 10^10^ OMV/well in fivefold serial dilutions. *E. coli* LPS (Invivogen, USA) at 10 µg/mL was used as a positive control according to the manufacturer’s instructions. Following 20 h of incubation, 50 µL of supernatant was removed from each well and added to a clean 96-well plate with 150 µL of Quanti-Blue (Invivogen, USA). Alkaline phosphatase activity was determined at 620 nm on a spectrophotometer.

#### Cytokine Production Assays

THP-1 cells [monocyte, M(naïve), and M(IFNγ)] were incubated with OMVs for 60 min as per OMV to cell binding assays (above). Following the 60 min incubation, culture fluid was removed, cell monolayers were washed with media, and incubated for a further 24 h in the absence of OMVs as described previously (4). After the second incubation period, supernatant was collected and centrifuged at 800 × *g* for 5 min at room temperature to remove remaining cells. Cytokine/chemokine secretion was measured using human TNF-α, IL-1β, IL-8, and IL-10 ELISA Kits (Jomar Life Research, VIC, Australia) according to the manufacturer’s instructions.

#### THP-1 and OMV Cytotoxicity Assays

THP-1 cells (both unprimed and primed with *P. gingivalis* OMVs at 100 ng protein/mL for 4 h) were treated with unlabeled OMVs in cell suspension at the cell concentration and OMV to cell ratios indicated above. OMV-induced cytotoxicity was determined following 4 h of OMV incubation by trypan blue exclusion using a Z1 Coulter Particle Counter (Beckman Coulter, NSW, Australia) as previously described (42).

#### *In Vitro* Inflammation Activation

##### THP-1 IL-1β ELISA

THP-1 cells were seeded in 96-well flat-bottom tissue culture plates at 1 × 10^5^ cells/well in 200 µL. Cells were primed with *E. coli* LPS (100 ng/mL), *P. gingivalis* LPS (100 ng/mL), *T. denticola* LPS (100 ng/mL), *T. forsythia* LPS (100 ng/mL), *P. gingivalis* OMVs (100 ng protein/mL), *T. denticola* OMVs (100 ng protein/mL), *T. forsythia* OMVs (100 ng protein/mL), or left unprimed for 4 h at 37°C in a 5% CO_2_ incubator. Cells were then stimulated with positive inflammasome activation controls Nigericin (10 µM) (Invivogen, USA), Silica (125 mg/mL) (US Silica, USA), or transfected with Poly(dAdT) (250 ng/mL) (Invitrogen, USA) using lipofectamine LTX (Invivogen, USA) as per the manufacturer’s instructions for a further 6 h. Primed cells were also treated with *P. gingivalis, T. denticola*, and *T. forsythia* OMVs at OMV: cell ratios of 10:1, 50:1, and 100:1 for 6 h or *E. coli, P. gingivalis, T. denticola*, and *T. forsythia* LPS (100 µg/mL) for 6 h. Cellular supernatants were collected and analyzed for IL-1β secretion by a human IL-1β ELISA Kit (Jomar Life Research, VIC, Australia) according to the manufacturer’s instructions.

##### THP-1 ASC Antibody Flow Cytometry and Imaging

Inflammasome formation was confirmed visually and by flow cytometry using ASC-specific antibody ASC (N-15)-R: sc-22514-R (Santa Cruz Biotechnology) and secondary antibody Alexa Fluor 488 goat anti-rabbit IgG (Santa Cruz Biotechnology) as previously described (43, 44). Briefly, THP-1 monocytes, M(naïve), and M(IFNγ)-differentiated cells were primed with *P. gingivalis* OMVs (100 ng protein/mL) for 4 h and then gently removed mechanically from culture plates using 15 mL PBS through a 10 mL pipet equipped with a 21 G needle (Terumo). Cells were spun (400 × *g* × 5 min) and resuspended in 500 µL RPMI 1640 medium (Sigma) without HI-FCS. THP-1 cells were then treated with *P. gingivalis, T. denticola*, and *T. forsythia* OMVs for 45 min, fixed with 2 mL of 100% ethanol for 15 min, and immunostained with primary and secondary antibodies.

Stained cells were assessed by flow cytometry using an appropriate laser for the excitation and filter sets for emission of Alexa Fluor 488. Data were analyzed first by creating a scatter gate around the main cell population (SSC-area versus FSC-area dot plot) to exclude debris and outlying events. Scatter gate events were then singlet gated to exclude cell doublets (FSC-width versus FSC-area dot plot). Singlet gate events were plotted by whole cell fluorescence (using empty channel BV 421-A) and ASC (FITC)-area, to observe increased in ASC fluorescence.

To assess fluorescent ASC accumulation by microscopy, fixed and ASC antibody-labeled cells were further stained with DAPI as previously described (45) to visualize cell DNA. Stained cells were stored in diamond antifade (Life Technologies) overnight and imaged on an OMX V4 Blaze super-resolution microscope (Deltavision) to observe fluorescent ASC specks.

##### IL-1β ELISA of Immortalized Bone Marrow-Derived Macrophage (IBMDM) Knockout Cell Lines

Specific inflammasome activation pathways were determined using wild-type, Caspase 1, ASC, NLRP3, and AIM2 knockout IBMDM cell lines. IBMDM cells were cultured in complete DMEM (10% v/v fetal calf serum, 3.5% v/v Glucose, 1% v/v Pen/Strep, and 1% v/v l-Glut) in a 5% CO_2_ incubator at 37°C. Cells were seeded in 96-well flat-bottom tissue culture plates at 1 × 10^5^ cells/well in 200 µL, primed with *E. coli* LPS (100 ng/mL) 4 h at 37°C in a 5% CO_2_ incubator, and 6-h inflammasome activation assays performed as above.

##### IL-1β Western Blot

THP-1 inflammasome assays were performed as above with the following secondary activating signals; positive control Nigericin and *P. gingivalis, T. denticola*, and *T. forsythia* OMVs for 6 h. Cellular supernatants were collected and the remaining cells washed with PBS and lysed using 100 µL Cell Lysis Buffer [15 mL MilliQ with 0.5% w/v SDS, 0.05 M TrisCl, 1 mM dithiothreitol, 0.5% Triton X, and 1 Complete Mini Protease Inhibitor Cocktail Tablet (Roche Diagnostics, Germany)] on ice for 30 min and centrifuged 600 × *g* × 10 min to collect the resulting supernatant. Cell lysate or supernatant samples (10 µg) were precipitated for SDS-PAGE, as previously described (46) by addition of trichloroacetic acid to a final concentration of 10% (v/v) and incubated 20 min at 4°C. Precipitated proteins were collected by centrifugation (10 min, 16,000 × *g*), resuspended in 20 µl reducing sample buffer [10% (w/v) SDS, 0.05% (w/v) bromophenol blue, 25% (v/v) glycerol, and 0.05% (v/v) 2-mercaptoethanol], and the pH adjusted with the addition of 10 µl of 1.5 M Tris/HCl, pH 8.0, and then heated for 5 min at 100°C, prior to loading on to precast 12% v/v acrylamide gels. Western blots were performed as previously described (47). Briefly, the primary antibody Anti-IL-1β Armenian Hamster IgG (eBioScience) was used at a 1:1,000 dilution, secondary antibody Anti-Arm Hamster IgG Biotin (eBioScience) was used at a 1:2,000 dilution, and Avidin-HRP (Thermo Fisher Scientific, SA, Australia) was used at a 1:2,000 dilution. The proteins determined by Western blot analysis were compared to purified IL-1β (Jomar Life Research, VIC, Australia) controls included on each gel and identified on the basis of MW.

#### *In Vivo* Inflammasome Activation

All animal experimental procedures were carried out in strict accordance with the recommendations in the *Australian Code of Practice for the Care and Use of Animals for Scientific Purposes*. The protocols for the experiments were approved by The University of Melbourne Ethics Committee for Animal Experimentation (approval number 1212363). Mice (6–8 weeks) C57BL/6 J were obtained from Department of Medicine, Royal Melbourne Hospital, The University of Melbourne, Parkville, VIC, Australia.

To evaluate the priming efficacy of *P. gingivalis* OMVs *in vivo* 38 mice were divided into three groups; the control group (*N* = 6) that received an intraperitoneal injection of PBS (100 µL), the LPS-primed group (*N* = 16) that received an intraperitoneal injection of *E. coli* LPS (100 µL of 1 µg/mL), and the OMV-primed group (*N* = 16) that received an intraperitoneal injection of *P. gingivalis* OMVs (100 µL of 1 µg protein/mL). Following 72 h the control group received a second intraperitoneal injection of PBS. The LPS- and OMV-primed groups were divided into four subgroups to be treated with nigericin, silica, OMVs, or left untreated. Untreated subgroups, LPS-Untreated (*N* = 4), and OMV-Untreated (*N* = 4) received an intraperitoneal injection of PBS (100 µL). Nigericin-treated subgroups, LPS-Nigericin (*N* = 4) and OMV-Nigericin (*N* = 4), received an intraperitoneal injection of nigericin (100 µL of 0.2 mM). Silica-treated subgroups, LPS-Silica (*N* = 4) and OMV-Silica (*N* = 4), received an intraperitoneal injection of silica (100 µL of 0.5 mg/mL). OMV-treated subgroups, LPS-OMV (*N* = 4) and OMV-OMV (*N* = 4), received an intraperitoneal injection of *P. gingivalis* OMVs (100 µL of 1 mg protein/mL). Fifteen minutes post injection mice were killed and intraperitoneal cells harvested with 5 mL PBS. Intraperitoneal cells were collected by centrifugation (600 × *g* × 10 min at room temperature) and resuspended in 1 mL of RPMI 1640 medium without HI-FCS.

##### Immune Cell Counts and Composition of Intraperitoneal Washes

Intraperitoneal washes were analyzed by flow cytometry using fluorescent antibodies against F4/80 and CD11b, Ly6G, CD11c, TCRb, and CD19 to identify macrophage (F4/80 and CD11b), neutrophil (Ly6G), dendritic cell (CD11c), T cell (TCRb), and B cell (CD19) populations, respectively. Total cell counts were determined using a Z1 Coulter Particle Counter (Beckman Coulter, NSW, Australia) and hemocytometer.

##### IL-1β Secretion by *In Vitro* Primed and *Ex Vivo* Activated Intraperitoneal Macrophages

Following centrifugation and resuspension in complete DMEM, intraperitoneal samples were incubated for 6 h at 37°C. Intraperitoneal cells from naive mice (unprimed and unactivated) were also cultured overnight at 37°C in a 5% CO_2_ incubator on 96-well tissue culture plates to allow the adherence of macrophages. Unbound cells were removed and the adhered macrophages primed for 4 h with *P. gingivalis* OMVs (100 ng protein/mL) then stimulated with nigericin, silica, *P. gingivalis, T. denticola*, or *T. forsythia* OMVs at OMV:cell ratios of 10:1, 50:1, and 100:1 for 6 h. Cellular supernatants of intraperitoneal samples and cultured macrophages were collected and analyzed for IL-1β secretion by a human IL-1β ELISA Kit (Jomar Life Research, VIC, Australia) according to the manufacturer’s instructions.

##### *In Vivo* Intraperitoneal Cell Inflammasome Detection by ASC Antibody Flow Cytometry and Microscopy

Inflammasome formation was confirmed by flow cytometry using ASC-specific antibody ASC (N-15)-R: sc-22514-R as above for THP-1 cells (see THP-1 ASC Antibody Flow Cytometry and Imaging) and described previously (43, 44). Washes were additionally stained with F4/80 and CD11b to correlate ASC inflammasome specks with macrophage populations.

### Statistical Analysis

The abovementioned methods were statistically analyzed using Student’s *t*-test with a minimum size of three biological replicates with three technical replicates. Significant differences were determined as *p* < 0.05. Error bars represent the SEM of each data subset.

## Results

### Host Monocytes and Macrophages Bind and Phagocytose Periodontal OMVs

**Figure 1:**
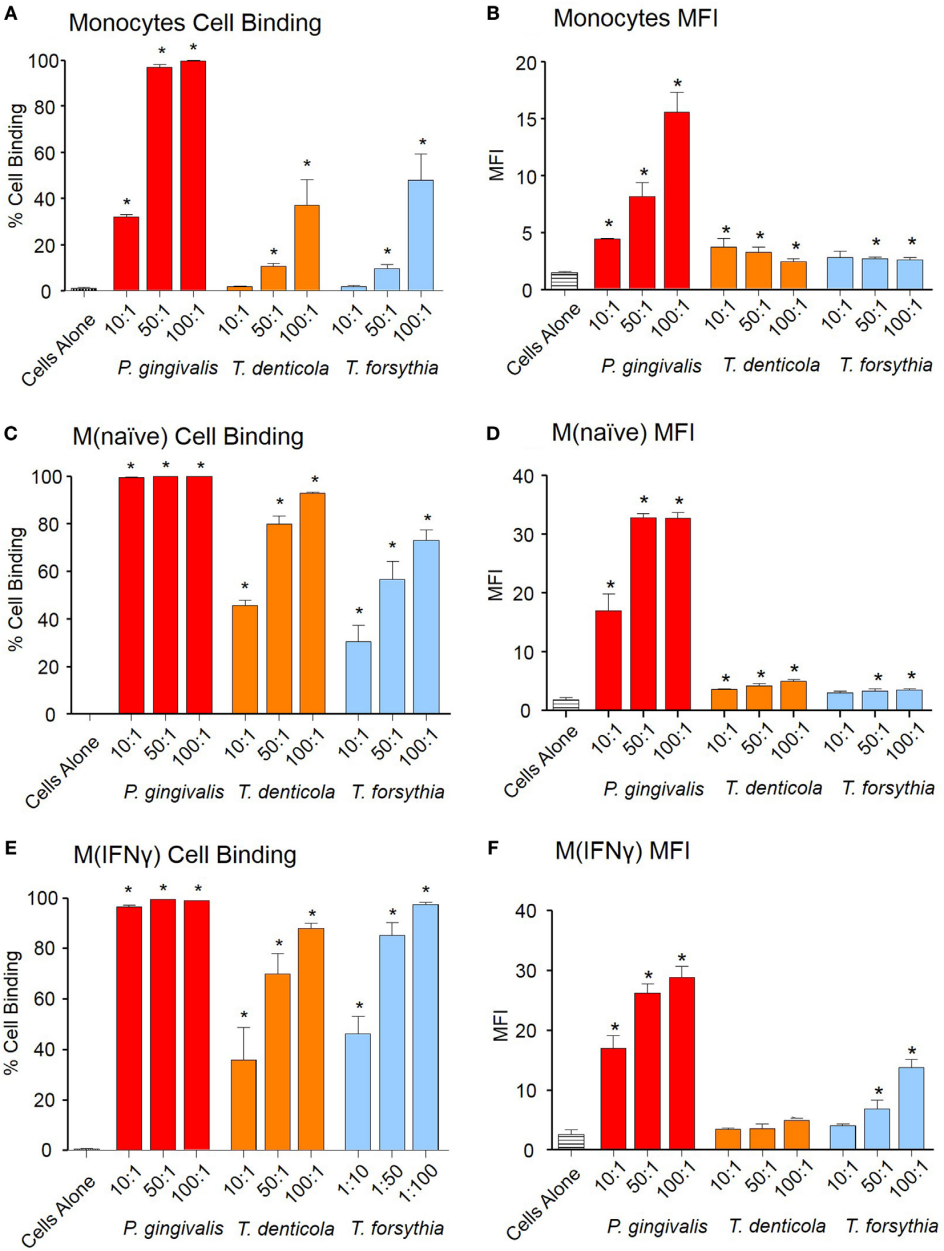
Periodontal pathogen outer membrane vesicles (OMVs) bind THP-1 cell subsets. Cell binding assays were performed with THP-1 cell suspensions of monocytes and monolayers of M(naïve) and M(IFNγ) macrophages incubated with PKH26-labeled *Porphyromonas gingivalis, Treponema denticola*, and *Tannerella forsythia* OMVs at three OMV to cell ratios (10:1, 50:1, and 100:1). Cells were incubated for 60 min, washed, and OMV binding determined by flow cytometry. Results are expressed as the percentage of THP-1 cells with at least one bound OMV **(A,C,E)** or the mean fluorescence intensity (MFI) of each sample **(B,D,F)**, which indicates the quantity of OMVs bound per cell. Data are represented as mean ± SEM of three replicates. *represents a result significantly higher (*p* < 0.05) than the fluorescence of untreated cells.

To investigate early OMV interactions with THP-1 monocytes, M (naïve), and differentiated M (IFNγ) macrophages, OMVs from *P. gingivalis, T. denticola*, and *T. forsythia* were labeled with lipid fluorescent dye PKH26 and binding determined by flow cytometry. Periodontal bacteria OMVs were found to bind to monocytes, PMA-treated M(naïve), and cytokine-treated M(IFNγ) macrophages in a dose-dependent manner (Figure 1). *P. gingivalis* OMVs bound more cells in higher numbers [as determined by mean fluorescence intensity (MFI)] than either *T. denticola* (*p* < 0.005) or *T. forsythia* OMVs (*p* < 0.01) (Figure 1). M(naïve) and M(IFNγ) macrophages were capable of binding OMVs with far greater affinity than undifferentiated monocytes (*p* < 0.01) (Figure 1). Of the three pathogens *P. gingivalis* OMVs demonstrated the greatest affinity for THP-1 cells, both in percentage cell binding (Figures 1A,C,E) and MFI (Figures 1B,D,F). While *T. denticola* and *T. forsythia* OMVs interacted with monocytes to a similar degree (Figure 1A), *T. denticola* OMVs were found to bind M(naïve) macrophages to a higher level (*p* < 0.05) (Figure 1C), while *T. forsythia* OMVs had higher binding to M(IFNγ) macrophages (*p* < 0.05) (Figure 1E). Both *T. denticola* and *T. forsythia* OMVs bound THP-1 cells poorly as indicated by MFI (Figures 1B,D,F).

**Figure 2:**
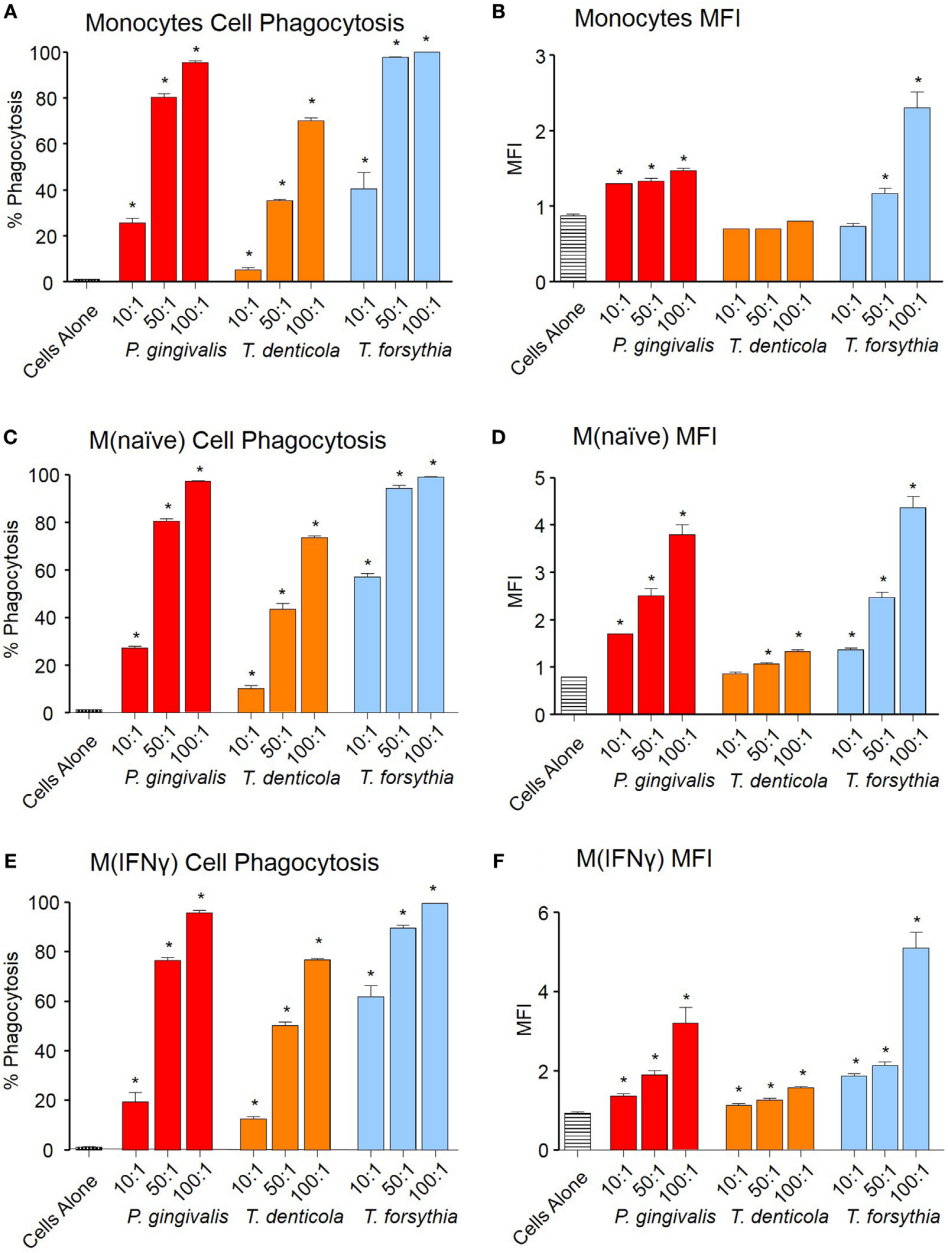
Periodontal pathogen outer membrane vesicles (OMVs) are phagocytosed by THP-1 cell subsets. Cell phagocytosis assays were performed with THP-1 cell suspensions (monocytes) and monolayers [M(naïve) and M(IFNγ)] incubated with pHrodo-labeled *Porphyromonas gingivalis, Treponema denticola*, and *Tannerella forsythia* OMVs at three OMV to cell ratios (10:1, 50:1, and 100:1). Cells were incubated for 60 min, washed, and OMV phagocytosis determined by flow cytometry. Results are expressed as the percentage of THP-1 cells with at least one bound OMV **(A,C,E)** or the mean fluorescence intensity (MFI) of each sample **(B,D,F)**, which indicates the quantity of OMVs bound per cell. Data are represented as mean ± SEM of three replicates. *represents a result significantly higher (*p* < 0.05) than the fluorescence of untreated cells.

Phagocytosis of OMVs was determined by labeling OMVs with the pH-sensitive dye pHrodo, which increases fluorescent intensity in the low pH conditions within a phagolysosome. OMVs from all periodontal pathogens were phagocytosed in a dose-dependent manner both in percentage cell binding (Figures 2A,C,E) and MFI (Figures 2B,D,F). While percentage phagocytosis was equal among cell types, MFI revealed M(naïve) and M(IFNγ) were more proficient at phagocytosis than monocytes; M(IFNγ) cells produced the highest MFI (*p* < 0.01), followed by M(naïve) cells (*p* < 0.01) (Figures 2B,D,E). *T. forsythia* OMVs were phagocytosed to the greatest degree (*p* < 0.001), as observed by both percentage phagocytosis and MFI, followed by *P. gingivalis* (*p* < 0.001) and *T. denticola* OMVs (*p* < 0.01) (Figure 2).

### OMVs from Periodontal Pathogens Differentially Activate THP-Blue NF-κB Reporter Cells

**Figure 3:**
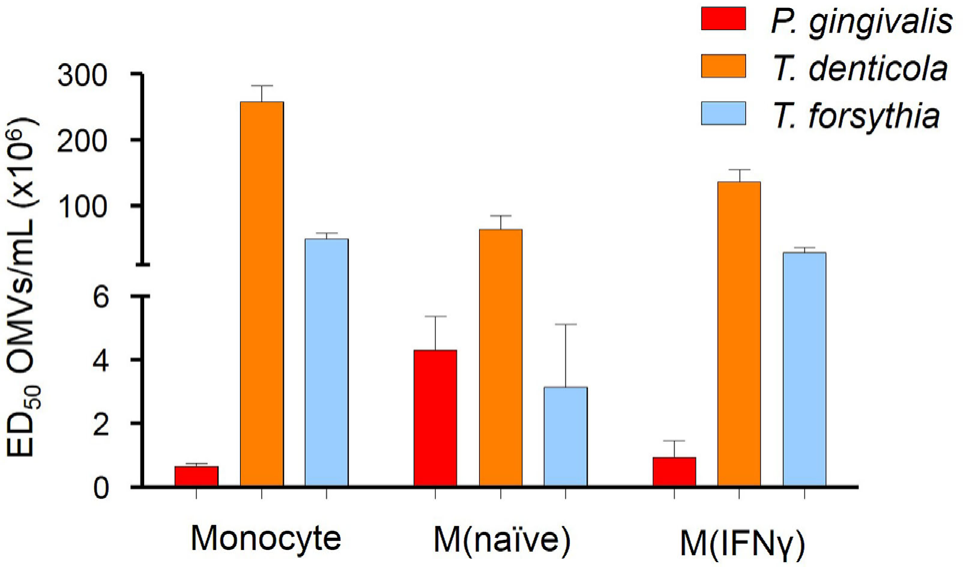
Periodontal pathogen outer membrane vesicles (OMVs) differentially activate NF-κB in THP-1-differentiated cells. THP-Blue cells [monocytes, M(naïve) and M(IFNγ) cell subsets] were incubated with either *Porphyromonas gingivalis, Treponema denticola*, or *Tannerella forsythia* OMVs (in fivefold dilutions) or positive control ligands Pam3CSK4 and lipopolysaccharide (LPS)-EB. Alkaline phosphatase secretion was determined with Quanti-Blue after 20 h incubation at 620 nm on a spectrophotometer. Data are represented as mean ± SEM of three biological replicates and results are presented as the number of OMVs required to achieve ED_50_. All Pam3CSK4 and LPS-EB controls gave positive results and were not included in the final data.

To determine if OMV binding induced NF-κB activation, a THP-Blue NF-κB activation reporter cell line was incubated with OMVs. THP-Blue are human monocytic cells stably expressing an NF-κB-inducible SEAP reporter construct. NF-κB was strongly activated by *P. gingivalis* OMVs (*p* < 0.001), moderately activated by *T. forsythia* OMVs (*p* < 0.01), and mildly activated by *T. denticola* OMVs (*p* < 0.05) (Figure 3). Significant differences were, however, observed between the activation of differentiated THP-Blue cells, by periodontal OMVs. *P. gingivalis* OMVs were potent activators of NF-κB in monocytes (*p* < 0.001) and M(IFNγ) macrophages (*p* < 0.001) compared to *T. denticola* and *T. forsythia* OMVs (Figure 3). *T. forsythia* OMVs had a lower ED50 (*p* < 0.01) than *T. denticola* for all THP-1 subsets tested (Figure 3). M(naive) macrophages were found to be equally activated by *P. gingivalis* OMVs and *T. forsythia* OMVs, which had significantly lower ED50s (*p* < 0.01) compared to *T. denticola* OMV NF-κB activation (Figure 3).

### Periodontal OMVs Induce Pro-inflammatory Cytokine Production in Host Macrophages

**Figure 4:**
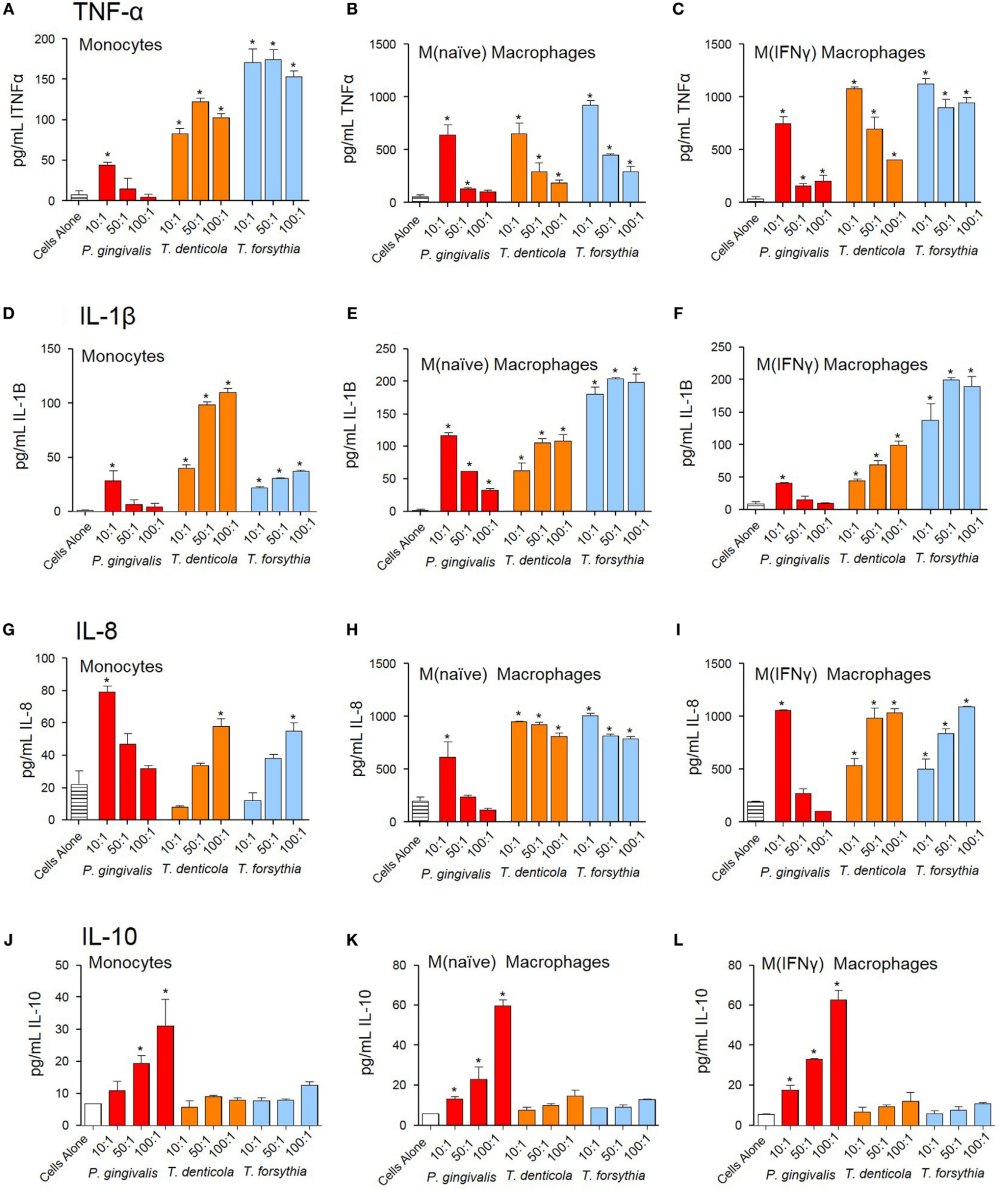
Periodontal pathogen outer membrane vesicles (OMVs) induce cytokine release from THP-1 cell subsets. THP-1 cytokine responses to *Porphyromonas gingivalis, Treponema denticola*, and *Tannerella forsythia* were determined following a 60 min incubation of OMVs (10:1, 50:1, and 100:1 OMV to cell ratios) on a THP-1 suspension (monocytes) or cell monolayer [M(naïve) and M(IFNγ)], followed by a 20-h incubation at 37°C. Results are expressed as picograms per milliliter of cytokine/chemokine TNFα **(A–C)**, IL-1β **(D–F)**, IL-8 **(G–I)**, and IL-10 **(J–L)** in the resulting supernatant. Data are represented as mean ± SEM of three replicates. *represents a significant (*p* < 0.05) increase from the cytokine secretion of unstimulated cells.

The NF-κB pathway has long been considered a key signaling pathway for the secretion of many cytokines and chemokines. We, therefore, examined how NF-κB activation by OMVs observed in monocytes, M(naïve), and M(IFNγ) cells translated to cytokine secretion. Undifferentiated monocytes, M(naïve), and M(IFNγ) cells were incubated with *P. gingivalis, T. denticola*, or *T. forsythia* OMVs and cytokine secretion determined by ELISA assays. Pro-inflammatory cytokines TNFα, IL-1β, and chemokine IL-8 were produced by monocytes, M(naïve), and M(IFNγ) cells after stimulation with all periodontal bacteria OMVs (*p* < 0.05) (Figures 4A–I). In all cases, M(naïve) and M(IFNγ) macrophages secreted the highest cytokine concentrations in response to OMVs; IL-8 and TNFα were produced in the highest concentrations followed by IL-1β secretion (Figure 4). In monocytes, *T. forsythia* OMVs induced the greatest TNFα secretion (*p* < 0.001) followed by *T. denticola* (p < 0.005) and *P. gingivalis* (*p* < 0.05) (Figure 4A). In M(naive) cells, TNFα responses to *T. denticola* and *T. forsythia* OMVs were very similar, while responses to *P. gingivalis* OMVs were significantly less at the 50:1 and 100:1 OMV to cell ratios (*p* < 0.05) (Figure 4B). M(IFNγ) cells incubated with *T. forsythia* OMVs secreted the greatest amount of TNFα, followed by *T. denticola* and *P. gingivalis* (Figure 4C). Interestingly, increasing concentrations of OMVs were observed to induce less TNFα secretion in all THP-1 cell subsets to all OMV stimuli (Figures 4A–C).

Similar to TNFα secretion, increasing *P. gingivalis* OMV concentrations induced decreasing IL-1β and IL-8 secretion in all host cell subsets, whereas increasing *T. denticola* and *T. forsythia* OMV concentrations incubated with host cells induced higher IL-1β and IL-8 secretion (Figures 4D–I). In monocytes, *T. denticola* induced the highest secretion of IL-1β, followed by *T. forsythia* and *P. gingivalis* (Figure 4D). M(naïve) macrophages incubated with *T. forsythia* OMVs secreted the highest amount of IL-1β, *T. denticola* OMVs (50:1 and 100:1) induced a similar response to *P. gingivalis* OMVs at 10:1, although the 50:1 and 100:1 ratios were significantly lower (*p* < 0.05) (Figure 4E). M(IFNγ) macrophages incubated with *T. forsythia* OMVs secreted the highest amount of IL-1β, followed by *T. denticola* and *P. gingivalis* (Figure 4F). Significant IL-8 secretion (*p* < 0.05) was only detected in monocytes at the highest *T. denticola* and *T. forsythia* OMV ratios (100:1) and the lowest *P. gingivalis* OMV ratio (10:1) (Figure 4G). In M(naïve) and M(IFNγ) macrophages, significant IL-8 was secreted after incubation with *T. denticola* and *T. forsythia* OMVs at all OMV to cell ratios but only by 10:1 *P. gingivalis* OMVs (Figures 4H,I).

*Porphyromonas gingivalis* OMVs were capable of inducing anti-inflammatory cytokine IL-10 in a dose-dependent manner (*p* < 0.05) at all OMV to cell ratios for M(naïve) and M(IFNγ) macrophages, but only the 50:1 and 100:1 ratios for monocytes (Figures 4J–L). *T. denticola* and *T. forsythia* OMVs did not induce IL-10 secretion at any OMV to cell ratio from any THP-1 subtype (Figures 4J–L).

### OMV-Induced Cytotoxicity Differs Between Unprimed and Primed THP-1 Cells

**Figure 5:**
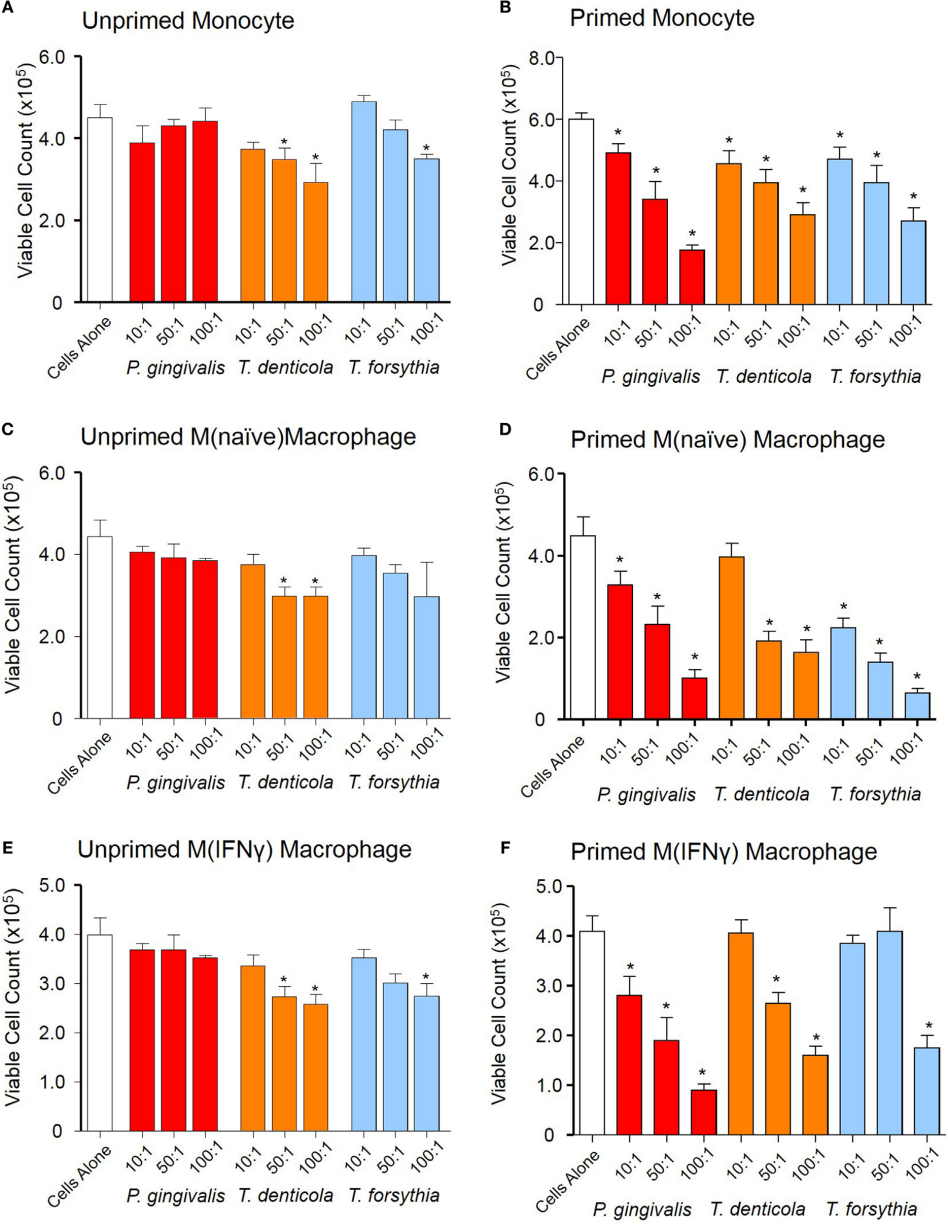
Periodontal outer membrane vesicle (OMV) cytotoxicity differs between unprimed and primed THP-1 cells. Cytotoxicity assays were performed on THP-1 monocytes, M(naïve), and M(IFNγ) macrophages left unprimed **(A,C,E)** or primed for 3 h with a low dose of *Porphyromonas gingivalis* OMVs **(B,D,F)**. Cells were treated with periodontal OMVs in increasing OMV to cell ratios (10:1, 50:1, and 100:1) in cell suspension for 4 h. Cell viability was determined by trypan blue exclusion and deteriorating cell counts determined by hemocytometer and a Z1 Coulter Particle Counter. Results are displayed as the number of remaining viable cells after 4 h. Data are represented as mean ± SEM of three replicates. *represents a significant (*p* < 0.05) decrease from the count of untreated cells.

Outer membrane vesicle-induced reduction of cell viability of THP-1 macrophages was observed by the exclusion of trypan blue and viable cell counts using a hemocytometer. Viable cell counts decreased in a dose-dependent manner when THP-1 cell suspensions [monocytes, M(naïve) and M(IFNγ)] were exposed to *T. denticola* OMVs (at 50:1 and 100:1 OMV to cell ratios) and *T. forsythia* OMVs (at the 100:1 ratio) (*p* < 0.05), while *P. gingivalis* OMVs were observed to have no cytotoxic effects under these conditions (Figures 5A,C,E).

We further investigated OMV-induced reduction of cell viability of primed THP-1 cell subsets. Upon incubation with *P. gingivalis, T. denticola*, and *T. forsythia* OMVs the primed THP-1 subsets had significantly (*p* < 0.05) less viable cells as compared to control and unprimed THP-1 cells (Figures 5B,D,F). In primed monocytes, all periodontal OMVs induced significant and comparable reductions in viable cells at the 10:1 and 50:1 ratios, while *P. gingivalis* OMVs induced the greatest viable cell reduction at 100:1 (*p* < 0.05) (Figure 5B). In primed M(naïve) macrophages, *T. forsythia* OMVs induced the greatest reduction in viable cells at all OMV to cell ratios, followed by *P. gingivalis* and *T. denticola* OMVs (Figure 5D). In primed M(IFNγ) macrophages, *P. gingivalis* OMVs stimulated a significant reduction in viable cells at all ratios; whereas *T. denticola* and *T. forsythia* OMVs stimulated significant viable cell decreases (*p* < 0.05) at 100:1 and 50:1 (Figure 5F). Intriguingly, there was no increase in apoptosis or necrosis in the unprimed or primed THP-1 cells upon incubation with any of the periodontal pathogen OMVs.

### Periodontal OMVs Induce Inflammasome Activation in THP-1 Monocytes/Macrophages

**Figure 6:**
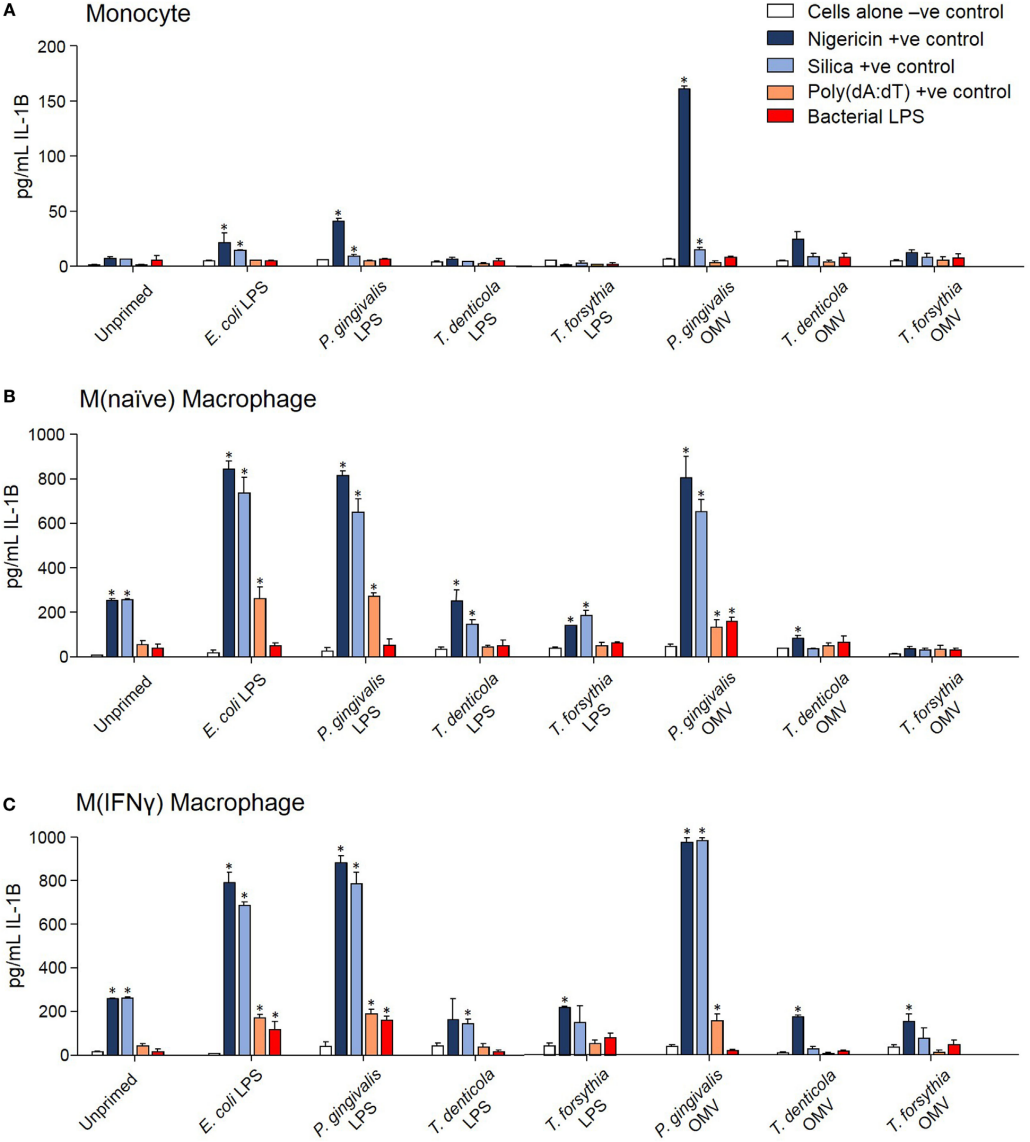
*Porphyromonas gingivalis* outer membrane vesicles (OMVs) and lipopolysaccharide (LPS) are priming agents for macrophage inflammasome activation. THP-1 monocytes **(A)**, M(naïve) **(B)**, and M(IFNγ) **(C)** macrophages were primed for 3 h with *Escherichia coli, P. gingivalis, Treponema denticola*, and *Tannerella forsythia* LPS and OMVs or left unprimed. Cells were then stimulated with positive controls nigericin, silica, and Poly(dA:dT) or *E. coli, P. gingivalis, T. denticola*, and *T. forsythia* LPS as required to match the priming material. Cellular supernatants were collected and IL-1β secretion detected by ELISA. Data are represented as mean ± SEM of three replicates. *represents a significant (*p* < 0.05) increase from the IL-1β secretion of untreated cells.

The capacity of periodontal OMVs to induce NF-κB activation, IL-1β secretion, and priming-dependent cell death in cytotoxicity assays was suggestive of inflammasome activation, we therefore explored the ability of OMVs to induce inflammasome activation in THP-1 cell subsets. Several NF-κB stimulating moieties were trialed for their capacity to prime THP-1 cell subsets prior to inflammasome activation. Priming THP-1 cells with *E. coli* LPS, *P. gingivalis* LPS, or *P. gingivalis* OMVs for 4 h was found to significantly increase (*p* < 0.05) nigericin-, silica-, and Poly(dA:dT)-induced IL-1β secretion from monocytes, M(naïve), and M(IFNγ) macrophages compared to unprimed cells (Figure 6). Priming with *T. denticola* and *T. forsythia* LPS and OMVs did not induce IL-1β secretion above unprimed cells (Figure 6). Poly(dA:dT) was found to be only an effective inflammasome activator for primed THP-1 monocytes, M(naïve) and M(IFNγ) cells (Figure 6). LPS although a strong inflammasome priming agent was found to be a weak activator for inflammasomes, with only high concentrations of LPS *E. coli* and *P. gingivalis* inducing inflammasome activation in primed M(IFNγ) cells M(naive) cells (Figures 6B,C). LPS extracted from *T. denticola* and *T. forsythia* was found to not be able to induce inflammasome activation (Figure 6).

**Figure 7:**
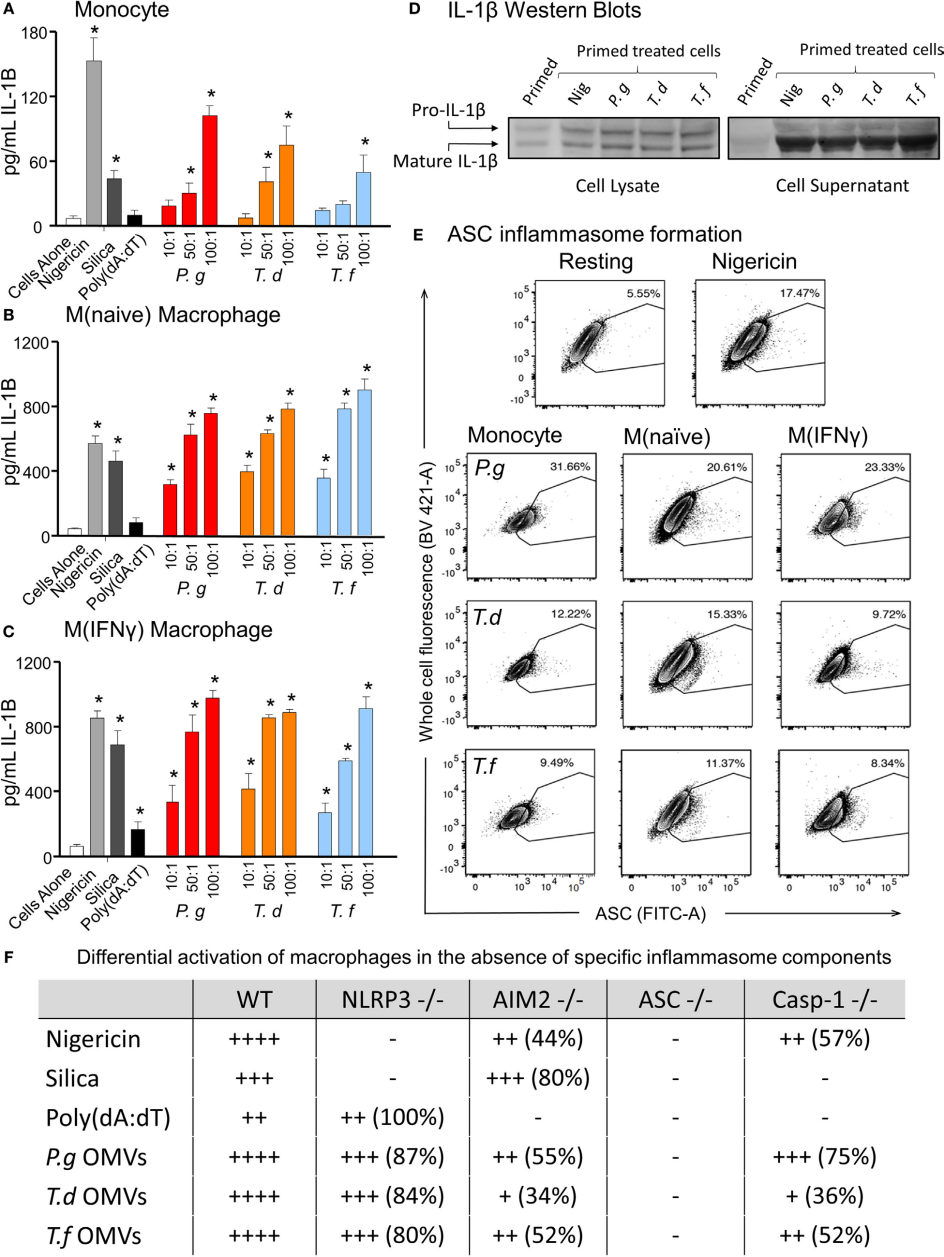
Periodontal outer membrane vesicles (OMVs) prime and activate inflammasome formation *in vitro*. THP-1 monocytes **(A)**, M(naïve) **(B)**, and M(IFNγ) **(C)** macrophages were primed with *Porphyromonas gingivalis* OMVs for 3 h then stimulated with either positive controls nigericin, silica, and Poly(dA:dT) or *P. gingivalis, Treponema denticola*, and *Tannerella forsythia* OMVs. Data are represented as mean ± SEM of three replicates. *represents a significant (*p* < 0.05) increase from the IL-1β secretion of untreated cells. **(D)** IL-1β western blots were performed on *P. gingivalis* OMV-primed (3 h) M(naïve) cell supernatant and cell lysate following treatment with nigericin, *P. gingivalis, T. denticola*, and *T. forsythia* OMVs. **(E)** ASC speck formation was detected by flow cytometry for THP-1 monocytes, M(naïve), and M(IFNγ) macrophages stimulated with *P. gingivalis, T. denticola*, and *T. forsythia* OMVs. OMV-induced increases in ASC fluorescence intensity were captured by the percentage of ASC+ cells over baseline background values, based on contour plots depicting ASC fluorescence (FITC-A) vs whole cell autofluorescence (BV421-A). Resting and nigericin controls displayed are from M(naïve) cells. **(F)** Wild-type immortalized bone marrow-derived macrophage (IBMDM) and NLRP3−/−, AIM2−/−, ASC−/−, and Caspase 1−/− knockout IBMDM strains were primed with *Escherichia coli* lipopolysaccharide (4 h) and treated with positive controls nigericin, silica, and Poly(dA:dT) or *P. gingivalis, T. denticola*, and *T. forsythia* OMVs. Results are presented as a qualitative indication (+ to ++++) of IL-1β secretion significantly greater (*p* < 0.05) than that of unstimulated IBMDM cells and a percentage decrease in IL-1β secretion as compared to secretion from wild-type IBMDM (see Figure 8).

In comparing unprimed cells, M(naïve) and M(IFNγ) macrophages produced 5–10-fold more IL-1β than monocytes (*p* < 0.001) (Figure 6). *P. gingivalis* LPS was found to be just as effective as *E. coli* LPS in priming all cell types for inflammasome activation, while *P. gingivalis* OMV priming resulted in a stronger nigericin-dependent response in monocytes (*p* < 0.001) and a weaker Poly(dA:dT) response in M (naive) cells (*p* < 0.05) (Figure 6). *P. gingivalis, T. denticola*, and *T. forsythia* OMVs all induced comparable dose-dependent inflammasome activation (as determined by IL-1β secretion) in *P. gingivalis* OMV-primed monocytes, M(naive), and M(IFNγ) macrophages (*p* < 0.05) (Figures 7A–C).

As pro-IL-1β can be released by dying cells in the absence of inflammasome activation and be detected as a false-positive signal by ELISA, IL-1β Western blots were performed to distinguish between pro-IL-1β (32 kDa) and mature IL-1β (17 kDa). Pro-IL-1β and mature IL-1β were found in the cell lysate of primed M (naive) cells and increased following nigericin, *P. gingivalis, T. denticola*, and *T. forsythia* OMV treatment (Figure 7D). Mature IL-1β was found in much higher concentrations, predominately in the cell supernatant, and was greatly increased following all OMV treatments (Figure 7D).

Outer membrane vesicle-induced inflammasome formation was further confirmed by utilizing a newly developed flow cytometry method to detect ASC speck formation in macrophage cells (44). A gating strategy was designed to capture whole cells undergoing ASC speck formation and to exclude pyroptotic bodies and non-cellular fluorescent debris. This gating strategy was adopted as it provided consistent results when applied to both *in vitro* THP-1 cells and *in vivo* peritoneal macrophages. Fixed and ASC antibody-stained THP-1 monocytes, M(naive), and M(IFNγ) cells were gated by cell size, cell singlets, and finally ASC antibody fluorescence (Whole Cell Fluorescence vs. ASC) to observe increases in the fluorescent signal indicative of ASC speck formation. Background ASC fluorescence [in M(naïve) cells] was recorded at approximately 5.55% and increased to 17.47% with the addition of positive control nigericin (Figure 7E). Increases in ASC fluorescence of 31.66, 12.22 and 9.49% were observed in monocytes stimulated with *P. gingivalis, T. denticola*, and *T. forsythia* OMVs, respectively (Figure 7E). *P. gingivalis* OMVs induced less ASC fluorescence in M(naïve) (20.61%) and M(IFNγ) (23.33%) macrophages compared with monocytes, whereas *T. denticola* and *T. forsythia* OMVs induced similar levels of speck formation in all cell types (Figure 7E).

**Figure 8:**
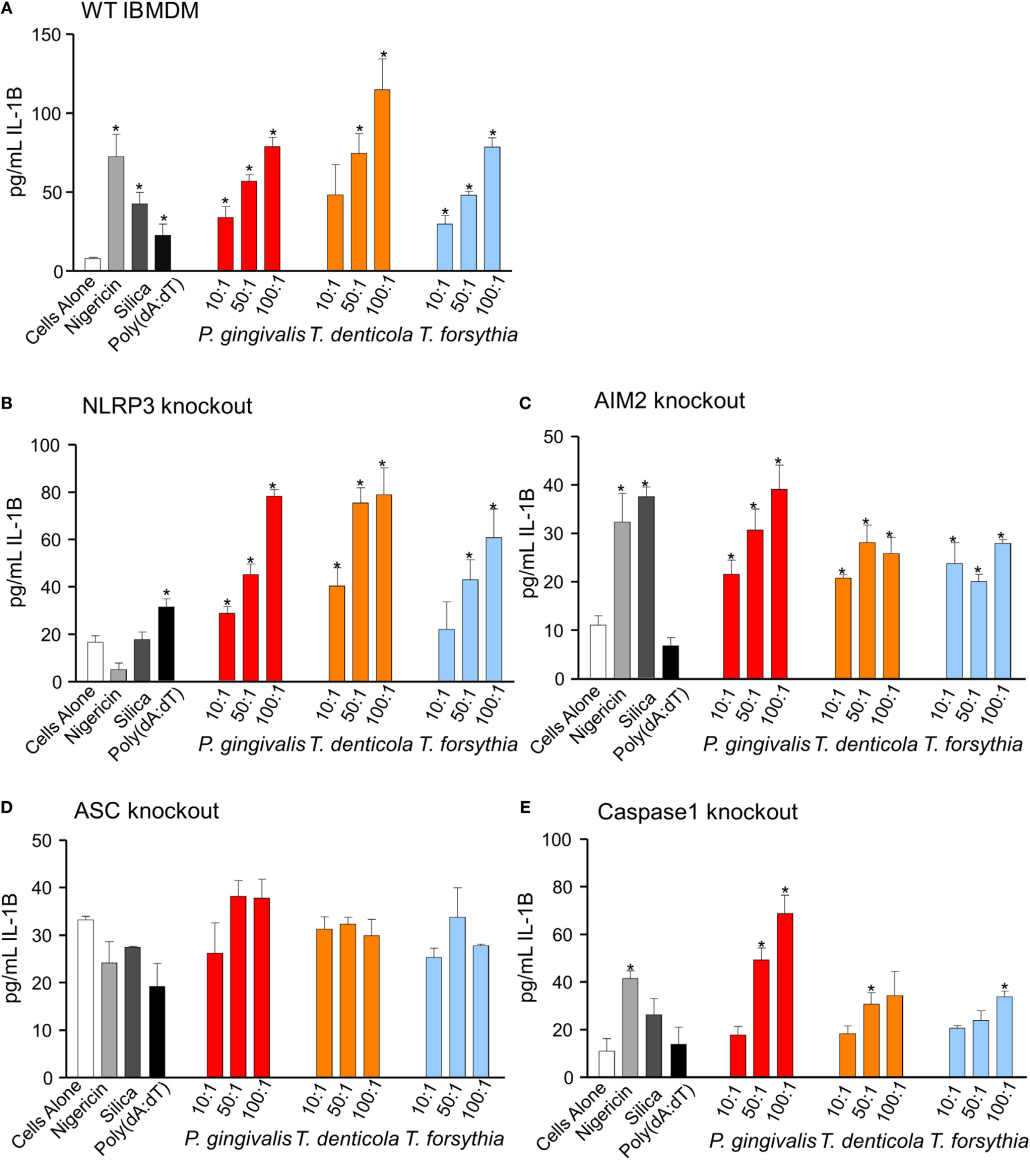
Inflammasome activation in immortalized bone marrow-derived macrophages (IBMDM) knockout cell lines. Wild-type IBMDM and NLRP3−/−, AIM2−/−, ASC−/−, and Caspase 1−/− knockout IBMDM strains were primed with *Porphyromonas gingivalis* outer membrane vesicles (OMVs, 3 h) and treated with positive controls nigericin, silica, and Poly(dA:dT) or *P. gingivalis, Treponema denticola*, and *Tannerella forsythia* OMVs at OMV:cell ratios of 10:1, 50:1, and 100:1 for 6 h. Cellular supernatants were collected and IL-1β secretion detected by an IL-1β ELISA Kit. Data are represented as mean ± SEM of three replicates. *represents a significant (*p* < 0.05) increase from the IL-1β secretion of untreated cells.

Immortalized bone marrow-derived macrophages generated from individual inflammasome gene-deficient mice (48) were used to identify the inflammasome activation pathways stimulated by periodontal OMVs. Wild-type IBMDMs were significantly stimulated (as determined by IL-1β secretion) by nigericin, silica, Poly(dA:dT), *P. gingivalis*, and *T. forsythia* OMVs at all OMV to cell ratios and *T. denticola* OMVs at the higher 50: 1 and 100: 1 ratios (*p* < 0.05) (Figures 7F and 8A). NLRP3−/− IBMDM cells were not activated by nigericin or silica controls but were activated by Poly(dA:dT), while *P. gingivalis, T. denticola*, and *T. forsythia* OMVs induced inflammasome activation similar to wild-type IBMDMs (Figures 7F and 8B). AIM2−/− IBMDMs did not respond to Poly(dA:dT) and were significantly less (*p* < 0.05) activated by *P. gingivalis, T. denticola*, and *T. forsythia* OMVs, nigericin and silica as compared to wild-type IBMDMs (Figures 7F and 8C). Importantly, ASC−/− IBMDMs were not activated by *P. gingivalis, T. denticola*, and *T. forsythia* OMVs or controls nigericin, silica, and Poly(dA:dT) (Figures 7F and 8D). Finally, Caspase 1−/− IBMDMs had significantly (*p* < 0.001) lower levels of inflammasome activation for *P. gingivalis, T. denticola*, and *T. forsythia* OMVs and controls compared to wild-type IBMDM cells (Figures 7F and 8E). Taken together, these results suggest that periodontal OMVs can activate ASC-dependent inflammasome complexes such as NLRP3 and AIM2.

### *P. gingivalis* OMVs Induce Inflammasome Activation *In Vivo*

**Figure 9:**
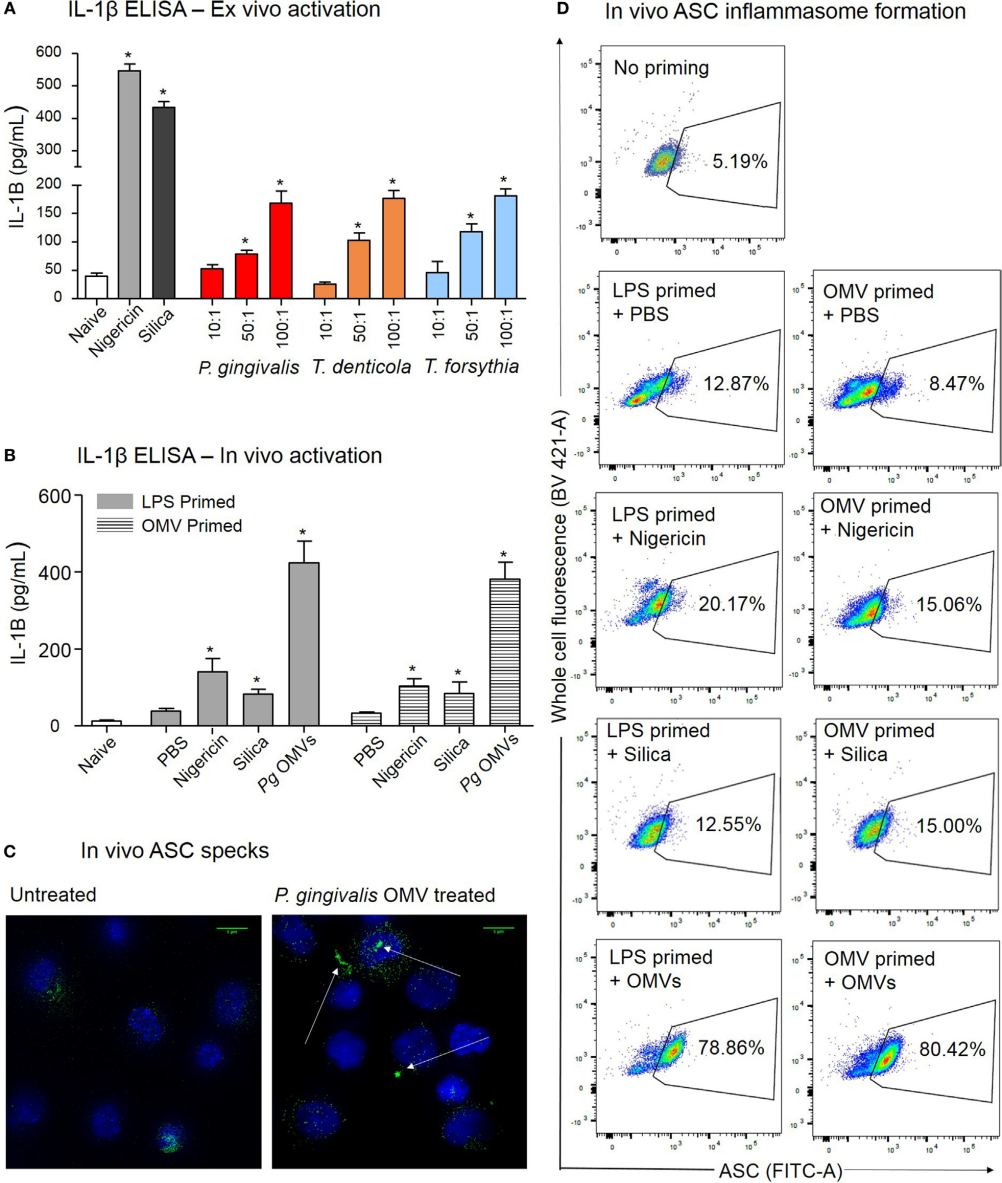
*Porphyromonas gingivalis* outer membrane vesicles (OMVs) prime and activate inflammasome formation *in vivo*. **(A)** Intraperitoneal cells from naive mice (unprimed and unactivated) were cultured overnight and stimulated *ex vivo* with nigericin, silica, *P. gingivalis, Treponema denticola*, or *Tannerella forsythia* OMVs, IL-1β secretion was determined by ELISA. **(B)** For *in vivo* inflammasome activation C57BL/6 J mice received intraperitoneal injections of phosphate-buffered saline (PBS, naïve), *Escherichia coli* lipopolysaccharide, or *P. gingivalis* OMVs 72 h prior to harvest to recruit immune cells to the peritoneal cavity. A second intraperitoneal injection of PBS, silica, nigericin, or *P. gingivalis* OMVs was administered 15 min prior to killing to active inflammasomes in peritoneal macrophages, IL-1β secretion was determined by ELISA. Data are represented as mean ± SEM of three replicates. *represents a significant (*p* < 0.05) increase from the IL-1β secretion of naive cells. **(C)** Microscopy was used to visually observe the dense accumulation of the inflammasome component ASC (green) in DAPI stained (blue) peritoneal macrophages primed and treated with *P. gingivalis* OMVs. **(D)** Inflammasome formation was confirmed by flow cytometry using ASC-specific antibody and macrophage markers (F4/80 and CD11b). For full gating strategy see Figure 10. Flow cytometry data display ASC-positive events plotted on a FITC area versus BV421 area (whole cell autofluorescence channel) dot plot.

Periodontal OMVs were capable of activating inflammasomes in THP-1 monocytes/macrophages, this virulence characteristic was further confirmed in primary murine macrophages extracted from intraperitoneal washes. Initially unprimed peritoneal macrophages were cultured overnight and primed *ex vivo* with *P. gingivalis, T. denticola*, and *T. forsythia* OMVs. Primary macrophages were found to have strong responses (determined by IL-1β secretion) after activation with nigericin and silica controls (*p* < 0.001) (Figure 9A). Activation with *P. gingivalis, T. denticola*, and *T. forsythia* OMVs all induced significant (*p* < 0.05) and comparable dose-dependent IL-1β secretion indicative of inflammasome activation (Figure 9A).

**Figure 10:**
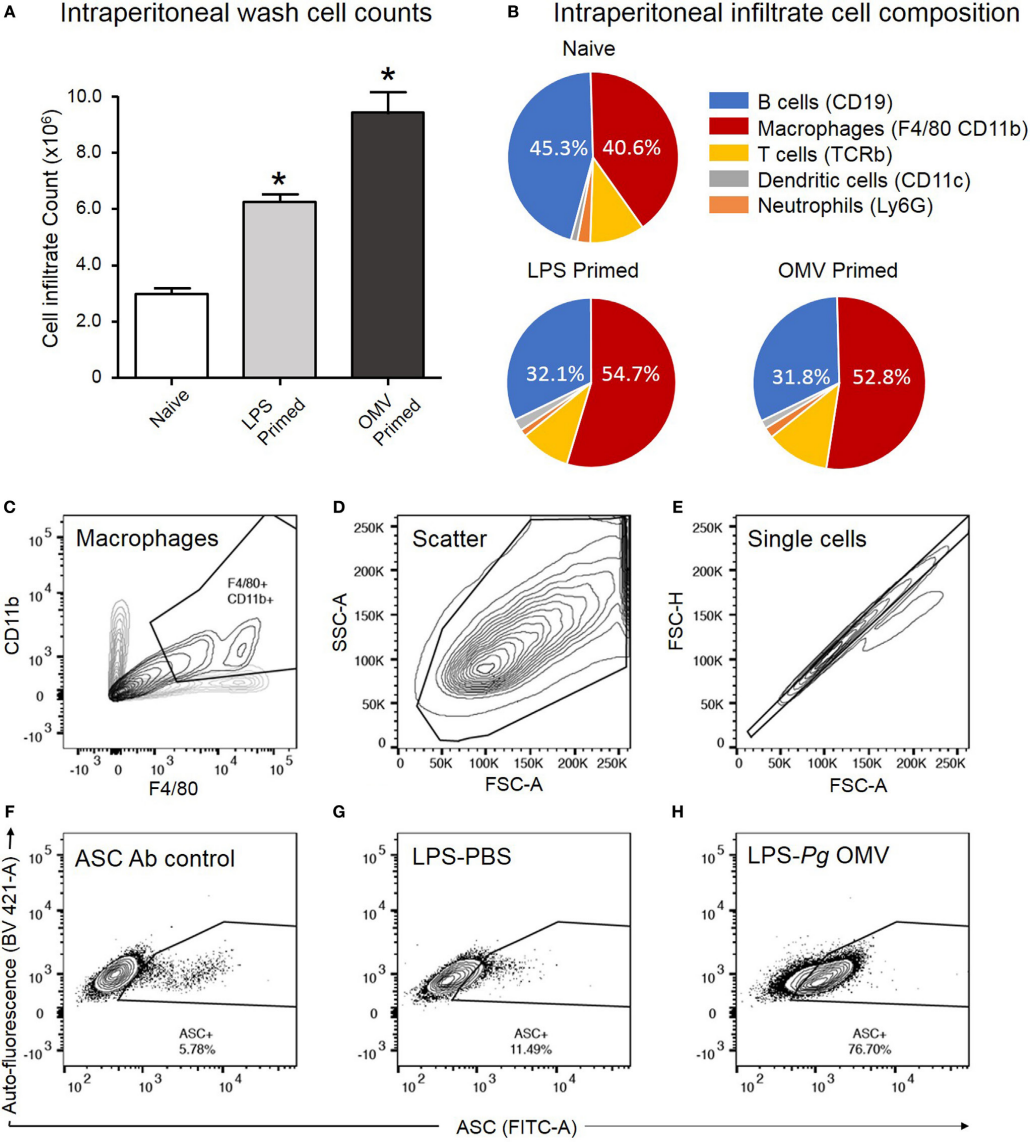
Analysis of ASC speck formation in peritoneal macrophages (*in vivo* activation). C57BL/6 J mice received intraperitoneal injections of phosphate-buffered saline (PBS, naïve), *Escherichia coli* lipopolysaccharide (LPS), or *Porphyromonas gingivalis* outer membrane vesicles (OMVs) 72 h prior to harvest to recruit immune cells to the peritoneal cavity. Intraperitoneal washes were counted using a Z1 Coulter Particle Counter to observe increase in cell recruitment following LPS and OMV priming **(A)**. Data are represented as mean ± SEM of three replicates. *represents a significant (*p* < 0.05) increase in cell counts from naïve washes. Following harvest intraperitoneal cells were analyzed by flow cytometry using fluorescent antibodies against F4/80 and CD11b, Ly6G, CD11c, TCRb, and CD19 to identify macrophage, neutrophil, dendritic cell, T cell and B cell populations, respectively **(B)**. Results in pie charts are expressed as the percentage of each detectable cell type in peritoneal samples and do not include cells outside these categories. Following a second intraperitoneal injection of PBS, nigericin, silica, or *P. gingivalis* OMVs, ASC speck formation in peritoneal macrophages was determined by flow cytometry. **(C)** CD11b- and F4/80-positive macrophages are selected from other peritoneal cells with a scatter gate. **(D)** Events within the scatter gate are plotted on a SSC-area versus FSC-area dot plot, the predominant population of ethanol-fixed cells are selected with a scatter gate. **(E)** Events within the scatter gate are plotted on a FSC-width versus FSC-area dot plot and single cells are selected with a singlet gate. **(F)** Single cells are plotted on a FITC-area versus BV421-area dot plot and a gate created to capture ASC fluorescence greater than that of the secondary antibody control. This population represent non-specific ASC antibody binding to peritoneal macrophages. **(G)** This population represents peritoneal cells which have been LPS primed *in vivo* but not activated. **(H)** This population represents peritoneal cells which have been LPS primed and *P. gingivalis* OMV activated. In this cell set diffuse ASC has condensed into single bright specks, increasing the detectable ASC fluorescence and indicating inflammasome activation. Data presented here were collected on a BD LSRFortessa X20 flow cytometer.

We further investigated the capacity of *P. gingivalis* OMVs to induce inflammasome activation *in vivo* by injecting *P. gingivalis* OMVs, *E. coli* LPS, or PBS into the peritoneal cavity of mice. Three days post injection the peritoneal cell infiltrate was harvested and characterized. *P. gingivalis* OMV-injected (primed) mice had significantly increased cellular infiltrate (cells/mL) compared to *E. coli* LPS-primed or PBS-injected mice (Figure 10A). Both *P. gingivalis* OMVs and *E. coli* LPS resulted in a similar cell population phenotype dominated by macrophages, which was significantly higher than PBS-injected mice (naive) (Figure 10B).

After 3 days of *P. gingivalis* OMV, *E. coli* LPS, or PBS priming, mice were injected with PBS, silica, nigericin, or *P. gingivalis* OMVs and 15 min later the peritoneal cells were harvested and inflammasome activation determined by IL-1β secretion. *In vivo* activation by *P. gingivalis* OMVs induced significantly (*p* < 0.01) higher IL-1β secretion than positive controls (nigericin and silica) in both LPS-primed and OMV-primed mice (Figure 9B). Nigericin, silica, and *P. gingivalis* OMVs all induced higher levels of IL-1β than PBS-injected mice (*p* < 0.05) (Figure 9B).

To confirm *in vivo* inflammasome activation by *P. gingivalis* OMVs, peritoneal cells were activated *in vivo* as above, immediately fixed and stained with ASC-specific antibody. ASC-speck formation was visualized by high-resolution fluorescent microscopy (Figure 9C). Only peritoneal cells primed and activated with *P. gingivalis* OMVs displayed distinct ASC speck formation, whereas a diffuse ASC fluorescence was observed in PBS-injected controls, indicative of no activation (Figure 9C and Video S1 in Supplementary Material). To quantify *in vivo* inflammasome activation, fixed and ASC antibody-stained peritoneal macrophages (F4/80+ and CD11b+) were analyzed for ASC-speck formation by flow cytometry (Figure 9D). Peritoneal cells were gated by F4/80 and CD11b fluorescence to identify macrophage populations, and Figures 10C–H describe the gating strategy to identify ASC speck-positive cells. *P. gingivalis* OMVs were found to induce ASC-speck formation in a significantly higher (*p* < 0.01) percentage of *E. coli* LPS (78.86% of cells) and *P. gingivalis* OMV (80.42% of cells) primed mice compared to PBS-, nigericin-, and silica-injected mice (ranging from 8.47 to 20.17% of cells) (Figure 9D).

## Discussion

Chronic inflammation plays a pivotal role in the progression of periodontal disease, yet the role of periodontal pathogen OMVs in inflammatory immune cells, such as macrophages, is understudied. Due to their nanoparticle size, adhesive, and proteolytic properties, OMVs are capable of migrating through host tissues, disrupting epithelial tight junctions, and delivering bacterial virulence factors to immune cells in underlying tissues (11). In this study, periodontal OMVs were found to interact strongly with monocytes and macrophages, inducing phagocytosis, NF-κB activation, cellular priming, and strong pro-inflammatory responses, including IL-1β secretion and inflammatory cell death *via* inflammasome activation both *in vitro* and *in vivo*.

*Porphyromonas gingivalis, T. denticola*, and *T. forsythia* OMVs were all found to induce significant and comparable inflammasome activation in primed, differentiated M(naïve), and M(IFNγ) macrophages (as observed by IL-1β secretion) while the activation of undifferentiated monocytes was approximately eightfold less. Although IL-1β ELISA assays are standard in quantifying the inflammasome activity of macrophages, not all IL-1β secretion can be ascribed purely to inflammasome activity, as pro-IL-1β can be released during apoptosis and detected extracellularly. IL-1β western blots confirmed the presence of mature IL-1β in the cell supernatant following OMV treatment and to a lesser extent pro-IL-1β in the cell lysate. Inflammasome activation was also confirmed visually by the localization of ASC fluorescence into the characteristic “speck” formation and *via* flow cytometry in macrophages. A combination of these techniques confirmed that *P. gingivalis, T. denticola*, and *T. forsythia* OMVs activate inflammasome responses in THP-1 monocytes, M(naive), and M(IFNγ) macrophages. We found that particular care must be taken with THP-1 cells during ASC antibody labeling, including the gentle, mechanical detachment of cells (as chemical detachment by trypsin-EDTA severely inhibited speck detection) and the use of FCS-free media during resuspension was critical.

Immortalized bone marrow-derived macrophage-knockout cell lines demonstrated that OMV-induced IL-1β secretion is dependent on the inflammasome component ASC but was only attenuated by the absence of Caspase 1, which may be attributed to its reported functional redundancy with Caspase 8 (26), or the non-canonical activation of Caspase 11 by intracellular LPS (49). *E. coli* OMVs have recently been shown to act as a delivery system for cytosolic LPS, which binds and activates cytosolic Caspase 11 (as well as Caspase 4 and 5) to trigger Caspase-1-independent pyroptosis through the cleavage of pore forming gasdermin D (50, 51). It is possible that Caspase 1−/− IBMDM cells utilize Caspase 11 to secrete IL-1β during OMV-induced inflammasome activation. However, the OMV/Caspase 11 activation pathway suggested by Vanaja et al. (51) is dependent upon the biological activity of OMV-associated LPS, as OMVs derived from an *E. coli* mutant lacking hexa-acylated lipid A (MKV15) induced greatly attenuated IL-1β secretion. *P. gingivalis, T. denticola*, and *T. forsythia* are known to possess atypical LPS which lacks functional hexa-acylated lipid A and therefore exhibits greatly reduced biological activity compared with *E. coli* LPS (52–54). We suggest that non-canonical activation of Caspase 11 by *P. gingivalis, T. denticola*, and *T. forsythia* LPS is unlikely to be the principle method of OMV-induced inflammasome activation, but is rather one of the many functionally redundant mechanisms through which periodontal pathogen OMVs may induce pyroptotic cell death and IL-1β secretion. AIM2 and (to a far lesser extent) NLRP3 knockout cell lines produced attenuated IL-1β secretion, which indicates that both inflammasome pathways contribute to OMV-induced inflammasome responses. NLRP3 and AIM2 have previously been suggested as the principle inflammasomes involved in *P. gingivalis*-induced inflammasome activation (31); however, it is possible that alternative inflammasome pathways, such as NLRP1 and NLRC4, may also play a role in OMV-induced IL-1β secretion in macrophages. Importantly, the absence of priming or key inflammasome machinery, such as ASC, abrogated IL-1β secretion in macrophage cells (IBMDMs), indicating that inflammasomes are principally responsible for IL-1β secretion in response to OMVs from each periodontal pathogen.

In investigating the ability of *P. gingivalis* OMVs to prime and activate inflammasomes *in vivo*, we observed that *P. gingivalis* OMVs were highly efficient at recruiting immune cells to the site of injection with a threefold increase over baseline cell numbers, compared to a twofold increase induced by *E. coli* LPS. Flow cytometry revealed that the phenotypic composition of intraperitoneal cells was predominately macrophages and B cells, most likely to be antibody-producing B1 cells due to their peritoneal location (55, 56). While B cells are capable of immune modulatory cytokine secretion, including IL-2, IL-4, IL-6, IL-10, IFNγ, and TNFα, they are reported to not possess inflammasome machinery and thus are likely minimal contributors to the IL-1β secretion observed from peritoneal cells (57). This was confirmed by flow cytometry where ASC speck formation occurred exclusively in the macrophage populations. *P. gingivalis* OMV-induced inflammasome activation was confirmed both *in vitro* and *in vivo* by ASC speck formation and strong IL-1β secretion from peritoneal macrophages. To the best of our knowledge, this is the first example of inflammasome activation by vesicles and clearly demonstrates the pro-inflammatory potential of *P. gingivalis* OMVs.

In addition to inflammasome induced IL-1β, periodontal pathogen OMVs were found to induce strong NF-κB activation and stimulate pro-inflammatory cytokine responses through secretion of TNFα and IL-8. PMA-differentiated M(naïve) and M(IFNγ) cells exhibited significantly higher NF-κB activation and cytokine secretion than THP-1 monocytes. This finding agrees with previous studies that have found M(naïve) and M(IFNγ) macrophages to have higher gene expression of NF-κB, TLR2, TLR7, and TLR8 as well as produce higher concentrations of pro-inflammatory (IL-1β, IL-6, and TNFα) and anti-inflammatory (IL-10) cytokines (58, 59) than monocytes. Gingival tissues of chronic periodontitis patients exhibit significantly higher levels of pro-inflammatory cytokines IL-1β, IL-6, IL-8, and TNFα than those of periodontally healthy subjects (60, 61), hence it is possible that periodontal pathogen OMVs are contributing to this response. The inflammatory effects of these cytokines have been extensively reviewed and include the activation of neutrophils, T and B lymphocytes, macrophages, natural killer cells, and osteoclasts to promote connective tissue destruction and alveolar bone resorption, the clinical hallmarks of chronic periodontitis (62–64). *P. gingivalis* OMVs were also found to induce the secretion of IL-10, a potent anti-inflammatory cytokine that is also significantly upregulated in the gingival crevicular fluid of periodontitis patients (65) and is known to suppress the synthesis of pro-inflammatory cytokines from various cell types (66, 67). While most OMV-induced cytokine responses increased in a dose-dependent manner, inverse cytokine responses were observed for *P. gingivalis* (TNFα, IL-1β, and IL-8), *T. denticola* (TNFα), and *T. forsythia* OMVs (TNFα), where cytokine secretion decreased with increasing OMV to cell ratios. As *P. gingivalis* OMVs were observed to stimulate NF-κB activation in THP-1 cells [to a greater degree in monocytes and M(IFNγ) macrophages than either *T. denticola* or *T. forsythia* OMVs] and the declining responses were not associated with an increase in cell death, this phenomenon may be an example of inflammatory anergy. Inflammatory anergy is a decreased or inhibited inflammatory response in monocytes and macrophages and is thought to be a protective mechanism to prevent detrimental inflammation in the presence of excessive LPS stimulation (68, 69). Also known as LPS tolerance, anergy is stimulated in monocytes by secondary contact with endotoxin and is generally associated with the upregulation of IL-10 synthesis (69), as observed for *P. gingivalis* OMVs in this study. Several studies have reported *P. gingivalis* OMV-mediated tolerance in monocyte/macrophage cell lines, in which pro-inflammatory responses (TNFα and IL-1β) to *E. coli* LPS or live *P. gingivalis* were greatly inhibited by previous exposure to OMVs (37, 70). Such differential responses benefit the host by minimizing the inflammatory damage induced by high OMV/bacterial concentrations and prolonged or repeated exposure, but may also benefit bacterial persistence by inhibiting bacterial clearance.

The prolonged presence of inflammatory cells may be a significant factor in chronic periodontal tissue destruction, as such several studies have examined the role of host cell death and its inhibition in periodontal disease (71). Four hours of stimulation with *P. gingivalis* OMVs induced no significant reduction in cell viability of THP-1 cells while *T. denticola* and *T. forsythia* OMVs induced minimal cell death of unprimed THP-1 populations. Under these conditions inflammasomes were not activated, hence low IL-1β secretion and minimal pyroptosis. OMV cytotoxicity was greatly increased by priming THP-1 cells with a low concentration of *P. gingivalis* OMVs. This is likely to be attributable to inflammasome-meditated pyroptosis, in which primed cells are stimulated by a second exposure to form caspase-dependent pores in the cellular membrane, which encourage inflammatory cell death. While previous studies have traditionally utilized *E. coli* LPS as a priming agent in inflammasome activation experiments, *P. gingivalis* LPS was found to be just as effective, despite its atypical LPS structure, while *T. denticola* and *T. forsythia* counterparts were found not to induce inflammasome priming. Interestingly, while *E. coli* and *P. gingivalis* LPS were adequate priming materials, they lacked the ability to provide a strong second signal and fully stimulate inflammasome activation, even when used at high concentrations. *P. gingivalis* OMVs were more effective than either LPS at priming THP-1 cells, and in addition were also capable of providing the second inflammasome activation signal. We suggest that either the particulate nature of OMVs is critical to inflammasome activation or the stimulation of multiple PRRs (39) has a synergistic effect greater than that of TLR4 stimulation alone. This finding has interesting implications for the role of OMVs in periodontal disease. The nanoparticle-like aspects of OMVs allow them to penetrate and disseminate through tissues, therefore, during infection an OMV concentration gradient will likely occur from the site of bacterial infection in the polymicrobial biofilm adhered to the tooth root. At distal areas from the initial site of infection OMV concentrations will be low enough to prime but not necessarily activate host cells, as disease bloom tissues will be primed by previous exposure to OMVs and the resulting immune responses will consequently be stronger.

All of these pro-inflammatory interactions are dependent upon initial OMV interactions with macrophages. OMVs derived from *P. gingivalis, T. denticola*, and *T. forsythia* were found to bind and be phagocytosed by M(naive)- and M(IFNγ)-differentiated THP-1 cells to a greater extent than monocytes; this increased capacity for phagocytosis has been observed in previous studies using whole bacterial cells (45, 72). A comparison of periodontal pathogen OMVs revealed that *P. gingivalis* OMVs bound all cell types with the greatest affinity, possibly due to the selective enrichment of gingipains on the vesicular surface (9). *P. gingivalis* gingipains contain adhesin domains known to aid coaggregation and cell binding (73, 74). However, *T. forsythia* OMVs were more readily phagocytosed than *P. gingivalis* or *T. denticola* OMVs in pHrodo phagocytosis assays. This may be attributable to the dependence of pHrodo fluorescence on acidification of the phagolysosome. *P. gingivalis* whole cells are known to evade phagosomal killing through several pathways including persisting in the cytoplasm and prevention of phagosome–lysosome fusion (75–77). If these mechanisms are shared by *P. gingivalis* OMVs it would render them partially undetectable by pHrodo but not by PKH-26 fluorescence.

Understanding the process of inflammation, and particularly the activity of key innate immune cells to bacterial virulence factors, is critical to understanding and modulating the progression of chronic periodontitis. This study observed the pathogenic potential of periodontal OMVs to both stimulate and inhibit macrophage pro-inflammatory responses *in vitro* and *in vivo*. While *T. denticola* and *T. forsythia* OMVs induced predominately pro-inflammatory responses, including TNFα, IL-1β, and IL-8 secretion in unprimed cells and strong inflammasome activation in primed THP-1 macrophages, *P. gingivalis* OMVs induced variable responses dependent on OMV concentration. Low concentrations of *P. gingivalis* OMV induced TNFα, IL-1β, and IL-8 secretion in unprimed cells, recruited inflammatory cells (*in vivo*), and primed macrophages to produce stronger immune responses, inducing both IL-1β secretion and pyroptosis upon second exposure. High concentrations of *P. gingivalis* OMVs were less inflammatory in unprimed cells due to LPS tolerance and inflammatory anergy but induced strong inflammasome activation upon second exposure. In the pathogenesis of periodontitis, *P. gingivalis* is generally regarded as the principal or keystone pathogen while *T. denticola* and *T. forsythia* are accessory pathogens playing a synergistic or supporting role (78–82). The potent but flexible immune stimulatory effects observed for *P. gingivalis* OMVs in this study are likely to assist whole cell *P. gingivalis* in manipulating and dysregulating the host immune response thereby initiating disease, while the pro-inflammatory effects of *T. denticola* and *T. forsythia* OMVs are likely to promote disease progression.

## Ethics Statement

All animal experimental procedures were carried out in strict accordance with the recommendations in the Australian Code of Practice for the Care and Use of Animals for Scientific Purposes. The protocols for the experiments were approved by The University of Melbourne Ethics Committee for Animal Experimentation (approval number 1212363).

## Author Contributions

Conception: JC, NO-S, JL, JH, AM, and ER. Design of the work: JC, NO-S, and AM. Acquisition and analysis: JC, WS, and AP-G. Drafting of work: JC. Writing—review and editing: all authors. Approval of final version: all authors. Accountability agreed: all authors.

## Conflict of Interest Statement

The authors declare that this research was conducted in the absence of any commercial or financial relationships that could be construed as a potential conflict of interest.

## Supplementary Material

Video S1 Video of ASC speck formation in peritoneal cells. Microscopy was used to visually observe the dense accumulation of the inflammasome component ASC (green) in DAPI-stained (blue) peritoneal macrophages primed and treated with *Porphyromonas gingivalis* outer membrane vesicles.

## References

[1] Haffajee AD Cugini MA Tanner A Pollack RP Smith C Kent RL Jr Subgingival microbiota in healthy, well-maintained elder and periodontitis subjects. J Clin Periodontol (1998) 25:346–53. 10.1111/j.1600-051X.1998.tb02454.x 9650869

[2] Socransky SS Haffajee AD Cugini MA Smith C Kent RL Jr. Microbial complexes in subgingival plaque. J Clin Periodontol (1998) 25:134–44. 10.1111/j.1600-051X.1998.tb02419.x 9495612

[3] Byrne SJ Dashper SG Darby IB Adams GG Hoffmann B Reynolds EC. Progression of chronic periodontitis can be predicted by the levels of *Porphyromonas gingivalis* and *Treponema denticola* in subgingival plaque. Oral Microbiol Immunol (2009) 24:469–77. 10.1111/j.1399-302X.2009.00544.x 19832799

[4] O’Brien-Simpson N Pathirana R Walker G Reynolds E. *Porphyromonas gingivalis* RgpA-Kgp proteinase-adhesin complexes penetrate gingival tissue and induce proinflammatory cytokines or apoptosis in a concentration-dependent manner. Infect Immun (2009) 77:1246–61. 10.1128/IAI.01038-08 19114547 PMC2643621

[5] Grenier D Mayrand D. Functional characterization of extracellular vesicles produced by *Bacteroides gingivalis*. Infect Immun (1987) 55:111–7. 3539799 10.1128/iai.55.1.111-117.1987 PMC260287

[6] Kuehn M Kesty N Bacterial outer membrane vesicles and the host–pathogen interaction. Genes Dev (2005) 19:2645–55. 10.1101/gad.1299905 16291643

[7] Haurat MF Aduse-Opoku J Rangarajan M Dorobantu L Gray MR Curtis MA Selective sorting of cargo proteins into bacterial membrane vesicles. J Biol Chem (2011) 286:1269–76. 10.1074/jbc.M110.185744 21056982 PMC3020734

[8] Nakao R Hasegawa H Ochiai K Takashiba S Ainai A Ohnishi M Outer membrane vesicles of *Porphyromonas gingivalis* elicit a mucosal immune response. PLoS One (2011) 6:e26163. 10.1371/journal.pone.0026163 22022548 PMC3193504

[9] Veith PD Chen YY Gorasia DG Chen D Glew MD O’Brien-Simpson NM *Porphyromonas gingivalis* outer membrane vesicles exclusively contain outer membrane and periplasmic proteins and carry a cargo enriched with virulence factors. J Proteome Res (2014) 13:2420–32. 10.1021/pr401227e 24620993

[10] Srisatjaluk R Doyle RJ Justus DE. Outer membrane vesicles of *Porphyromonas gingivalis* inhibit IFN-gamma-mediated MHC class II expression by human vascular endothelial cells. Microb Pathog (1999) 27:81–91. 10.1006/mpat.1999.0287 10458919

[11] Chi B Qi M Kuramitsu H. Role of dentilisin in *Treponema denticola* epithelial cell layer penetration. Res Microbiol (2003) 154:637–43. 10.1016/j.resmic.2003.08.001 14596901

[12] Sharpe SW Kuehn MJ Mason KM. Elicitation of epithelial cell-derived immune effectors by outer membrane vesicles of nontypeable *Haemophilus influenzae*. Infect Immun (2011) 79:4361–9. 10.1128/IAI.05332-11 21875967 PMC3257905

[13] Hajishengallis G. The inflammophilic character of the periodontitis-associated microbiota. Mol Oral Microbiol (2014) 29:248–57. 10.1111/omi.12065 24976068 PMC4232466

[14] Mathur A Michalowicz B Castillo M Aeppli D. Interleukin-1 alpha, interleukin-8 and interferon-alpha levels in gingival crevicular fluid. J Periodontal Res (1996) 31:489–95. 10.1111/j.1600-0765.1996.tb01414.x 8915952

[15] Lappin DF Kjeldsen M Sander L Kinane DF. Inducible nitric oxide synthase expression in periodontitis. J Periodontal Res (2000) 35:369–73. 10.1034/j.1600-0765.2000.035006369.x 11144410

[16] Hirose M Ishihara K Saito A Nakagawa T Yamada S Okuda K. Expression of cytokines and inducible nitric oxide synthase in inflamed gingival tissue. J Periodontol (2001) 72:590–7. 10.1902/jop.2001.72.5.590 11394393

[17] Masada MP Persson R Kenney JS Lee SW Page RC Allison AC. Measurement of interleukin-1 alpha and -1 beta in gingival crevicular fluid: implications for the pathogenesis of periodontal disease. J Periodontal Res (1990) 25:156–63. 10.1111/j.1600-0765.1990.tb01038.x 2141875

[18] Ishihara Y Nishihara T Kuroyanagi T Shirozu N Yamagishi E Ohguchi M Gingival crevicular interleukin-1 and interleukin-1 receptor antagonist levels in periodontally healthy and diseased sites. J Periodontal Res (1997) 32:524–9. 10.1111/j.1600-0765.1997.tb00568.x 9379320

[19] Martinon F Burns K Tshopp J. The inflammasome. A molecular platform triggering activation of inflammatory caspases and processing of proIL-beta. Mol Cell (2002) 10:417–26. 10.1016/S1097-2765(02)00599-3 12191486

[20] Netea MG Nold-Petry CA Nold MF Joosten LAB Opitz B van der Meer JHM Differential requirement for the activation of the inflammasome for processing and release of IL-1beta in monocytes and macrophages. Blood (2009) 113:2324–35. 10.1182/blood-2008-03-146720 19104081 PMC2652374

[21] Ellis TN Kuehn MJ. Virulence and immunomodulatory roles of bacterial outer membrane vesicles. Microbiol Mol Biol Rev (2010) 74:81–94. 10.1128/MMBR.00031-09 20197500 PMC2832350

[22] He Y Hara H Núñez G. Mechanism and regulation of NLRP3 inflammasome activation. Trends Biochem Sci (2016) 41:1012–21. 10.1016/j.tibs.2016.09.002 27669650 PMC5123939

[23] Veltman D Laeremans T Passante E Huber HJ. Signal transduction analysis of the NLRP3-inflammasome pathway after cellular damage and its paracrine regulation. J Theor Biol (2017) 415:125–36. 10.1016/j.jtbi.2016.12.016 28017802

[24] Adi Haji I Cellular Activation and Death in Response to Cytoplasmic DNA. Ph.D. thesis, The University of Queensland, Brisbane (2009).

[25] Fink S Cookson B. Caspase-1-dependent pore formation during pyroptosis leads to osmotic lysis of infected host macrophages. Cell Microbiol (2006) 8:1812–25. 10.1111/j.1462-5822.2006.00751.x 16824040

[26] Antonopoulos C Russo HM El Sanadi C Martin BN Li X Kaiser WJ Caspase-8 as an effector and regulator of NLRP3 inflammasome signaling. J Biol Chem (2015) 290:20167–84. 10.1074/jbc.M115.652321 26100631 PMC4536427

[27] Aglietti RA Estevez A Gupta A Ramirez MG Liu PS Kayagaki N GsdmD p30 elicited by caspase-11 during pyroptosis forms pores in membranes. Proc Natl Acad Sci U S A (2016) 113:7858–63. 10.1073/pnas.1607769113 27339137 PMC4948338

[28] Liu X Zhang Z Ruan J Pan Y Magupalli VG Wu H Inflammasome-activated gasdermin D causes pyroptosis by forming membrane pores. Nature (2016) 535:153–8. 10.1038/nature18629 27383986 PMC5539988

[29] Shi J Zhao Y Wang K Shi X Wang Y Huang H Cleavage of GSDMD by inflammatory caspases determines pyroptotic cell death. Nature (2015) 526(7575):660–5. 10.1038/nature15514 26375003

[30] Cunha LD Zamboni DS. Subversion of inflammasome activation and pyroptosis by pathogenic bacteria. Front Cell Infect Microbiol (2013) 3:76. 10.3389/fcimb.2013.00076 24324933 PMC3840304

[31] Park E Na HS Song YR Shin SY Kim YM Chung J. Activation of NLRP3 and AIM2 inflammasomes by *Porphyromonas gingivalis* infection. Infect Immun (2014) 82:112–23. 10.1128/IAI.00862-13 24126516 PMC3911849

[32] Das A Sinha M Datta S Abas M Chaffee S Sen C Review: monocyte and macrophage plasticity in tissue repair and regeneration. Am J Pathol (2015) 185:2596–606. 10.1016/j.ajpath.2015.06.001 26118749 PMC4607753

[33] Benoit M Desnues B Mege JL. Macrophage polarization in bacterial infections. J Immunol (2008) 181:3733–40. 10.4049/jimmunol.181.6.3733 18768823

[34] Lam R O’Brien-Simpson N Lenzo J Holden J Brammar G Walsh K Macrophage depletion abates *Porphyromonas gingivalis* induced alveolar bone resorption in mice. J Immunol (2014) 193:2349–62. 10.4049/jimmunol.1400853 25070844

[35] Imayoshi R Cho T Kaminishi H. NO production in RAW264 cells stimulated with *Porphyromonas gingivalis* extracellular vesicles. Oral Dis (2011) 17:83–9. 10.1111/j.1601-0825.2010.01708.x 20646228

[36] Qi MS Miyakawa H Kuramitsu HK. *Porphyromonas gingivalis* induces murine macrophage foam cell formation. Microb Pathog (2003) 35:259–67. 10.1016/j.micpath.2003.07.002 14580389

[37] Duncan L Yoshioka M Chandad F Grenier D. Loss of lipopolysaccharide receptor CD14 from the surface of human macrophage-like cells mediated by *Porphyromonas gingivalis* outer membrane vesicles. Microb Pathog (2004) 36:319–25. 10.1016/j.micpath.2004.02.004 15120158

[38] Friedrich V Gruber C Nimeth I Pabinger S Sekot G Posch G Outer membrane vesicles of *Tannerella forsythia*: biogenesis, composition, and virulence. Mol Oral Microbiol (2015) 30:451–73. 10.1111/omi.12104 25953484 PMC4604654

[39] Cecil JD O’Brien-Simpson NM Lenzo JC Holden JA Chen YY Singleton W Differential responses of pattern recognition receptors to outer membrane vesicles of three periodontal pathogens. PLoS One (2016) 11:e0151967. 10.1371/journal.pone.0151967 27035339 PMC4818014

[40] Rosen G Sela MN Naor R Halabi A Barak V Shapira L. Activation of murine macrophages by lipoprotein and lipooligosaccharide of *Treponema denticola*. Infect Immun (1999) 67:1180–6. 10024558 10.1128/iai.67.3.1180-1186.1999 PMC96444

[41] Pathirana RD O’Brien-Simpson NM Visvanathan K Hamilton JA Reynolds EC. Flow cytometric analysis of adherence of *Porphyromonas gingivalis* to oral epithelial cells. Infect Immun (2007) 75:2484–92. 10.1128/IAI.02004-06 17339349 PMC1865753

[42] Lenzo JC O’Brien-Simpson NM Orth RK Mitchell HL Dashper SG Reynolds EC Porphyromonas gulae has similar virulence and immunological characteristics to the human periodontal pathogen *Porphyromonas gingivalis*. Infect Immun (2016) 84:2575–85. 10.1128/IAI.01500-15 27354442 PMC4995892

[43] Sester DP Thygesen SJ Sagulenko V Vajjhala PR Cridland JA Vitak N A novel flow cytometric method to assess inflammasome formation. J Immunol (2015) 194:455–62. 10.4049/jimmunol.1401110 25404358

[44] Sester DP Zamoshnikova A Thygesen SJ Vajjhala PR Cridland SO Schroder K Assessment of inflammasome formation by flow cytometry. Curr Protoc Immunol (2016) 114:1–29. 10.1002/cpim.13 27479658

[45] Lam RS O’Brien-Simpson NM Holden JA Lenzo JC Fong SB Reynolds EC. Unprimed, M1 and M2 macrophages differentially interact with *Porphyromonas gingivalis*. PLoS One (2016) 11:e0158629. 10.1371/journal.pone.0158629 27383471 PMC4934774

[46] Pathirana RD O’Brien-Simpson NM Veith PD Riley PF Reynolds EC. Characterization of proteinase-adhesin complexes of *Porphyromonas gingivalis*. Microbiology (2006) 152:2381–94. 10.1099/mic.0.28787-0 16849802

[47] Dashper SG O’Brien-Simpson NM Bhogal PS Franzmann AD Reynolds EC. Purification and characterization of a putative fimbrial protein/receptor of *Porphyromonas gingivalis*. Aust Dent J (1998) 43:99–104. 10.1111/j.1834-7819.1998.tb06097.x 9612983

[48] Hornung V Bauernfeind F Halle A Samstad E Kono H Rock K Silica crystals and aluminum salts activate the NALP3 inflammasome through phagosomal destabilization. Nat Immunol (2008) 9:847–56. 10.1038/ni.1631 18604214 PMC2834784

[49] Kayagaki N Wong MT Stowe IB Ramani SR Gonzalez LC Akashi-Takamura S Noncanonical inflammasome activation by intracellular LPS independent of TLR4. Science (2013) 341:1246–9. 10.1126/science.1240248 23887873

[50] Kayagaki N Warming S Lamkanfi M Vande Walle L Louie S Dong J Non-canonical inflammasome activation targets caspase-11. Nature (2011) 479:117–21. 10.1038/nature10558 22002608

[51] Vanaja SK Russo AJ Behl B Banerjee I Yankova M Deshmukh SD Bacterial outer membrane vesicles mediate cytosolic localization of LPS and caspase-11 activation. Cell (2016) 165:1106–19. 10.1016/j.cell.2016.04.015 27156449 PMC4874922

[52] Schultz CP Wolf V Lange R Mertens E Wecke J Naumann D Evidence for a new type of outer membrane lipid in oral spirochete *Treponema denticola*: functioning permeation barrier without lipopolysaccharides. J Biol Chem (1998) 273:15661–6. 10.1074/jbc.273.25.15661 9624160

[53] Herath TDK Darveau RP Seneviratne CJ Wang C-Y Wang Y Jin L Tetra- and penta-acylated lipid A structures of *Porphyromonas gingivalis* LPS differentially activate TLR4-mediated NF-κB signal transduction cascade and immuno-inflammatory response in human gingival fibroblasts. PLoS One (2013) 8:e58496 10.1371/journal.pone.0058496 23554896 PMC3595299

[54] Posch G Andrukhov O Vinogradov E Lindner B Messner P Holst O Structure and immunogenicity of the rough-type lipopolysaccharide from the periodontal pathogen *Tannerella forsythia*. Clin Vaccine Immunol (2013) 20:945–53. 10.1128/CVI.00139-13 23616409 PMC3675976

[55] Haas KM Poe JC Steeber DA Tedder TF. B-1a and B-1b cells exhibit distinct developmental requirements and have unique functional roles in innate and adaptive immunity to *S. pneumoniae*. Immunity (2005) 23:7–18. 10.1016/j.immuni.2005.04.011 16039575

[56] Yenson V Baumgarth N. Purification and immune phenotyping of B-1 cells from body cavities of mice. Methods Mol Biol (2014) 1190:17–34. 10.1007/978-1-4939-1161-5_2 25015270

[57] Bao Y Cao X Review: the immune potential and immunopathology of cytokine-producing B cell subsets: a comprehensive review. J Autoimmun (2014) 55:10–23. 10.1016/j.jaut.2014.04.001 24794622

[58] Gmiterek A Kłopot A Wójtowicz H Trindade S Olczak M Olczak T. Immune response of macrophages induced by *Porphyromonas gingivalis* requires HmuY protein. Immunobiology (2016) 221:1382–94. 10.1016/j.imbio.2016.07.007 27473343

[59] Belfield L Interactions between Porphyromonas gingivalis and Macrophages in Oral Pathology [Dissertation]. University of Plymouth (2013).

[60] Tsai CC Ho YP Chen CC. Levels of interleukin-1 beta and interleukin-8 in gingival crevicular fluids in adult periodontitis. J Periodontol (1995) 66:852–9. 10.1902/jop.1995.66.10.852 8537867

[61] Yoshimura A Hara Y Kaneko T Kato I Secretion of IL-1 beta, TNF-alpha, IL-8 and IL-1a by human polymorphonuclear leukocytes in response to lipopolysaccharides from periodontopathic bacteria. J Periodontal Res (1997) 32:279–86. 10.1111/j.1600-0765.1997.tb00535.x 9138193

[62] Stashenko P Teles R D’Souza R. Periapical inflammatory responses and their modulation. Crit Rev Oral Biol Med (1998) 9:498–521. 10.1177/10454411980090040701 9825224

[63] Silva TA Garlet GP Fukada SY Silva JS Cunha FQ. Chemokines in oral inflammatory diseases: apical periodontitis and periodontal disease. J Dent Res (2007) 86:306–19. 10.1177/154405910708600403 17384024

[64] Graunaite I Lodiene G Maciulskiene V Pathogenesis of apical periodontitis: a literature review. J Oral Maxillofac Res (2011) 2:e1 10.5037/jomr.2011.2401 PMC3886078 24421998

[65] Bing X-J Sun X-J Shen G-H Xie H Ma M-Y Levels of IL-8, IL-10 in patients with chronic periodontitis and coronary heart disease. Shanghai Kou Qiang Yi Xue (2015) 24:598–601. 26598196

[66] Berg D Ralf K Rajewsky K Interleukin-10 is a central regulator of the response to LPS in murine models of endotoxic shock and the Schwartzman reaction but not to endotoxin tolerance. J Clin Invest (1995) 96:2339–47. 10.1172/JCI118290 7593621 PMC185885

[67] Zhang Q Chen B Yan F Guo J Zhu X Ma S Interleukin-10 inhibits bone resorption: a potential therapeutic strategy in periodontitis and other bone loss diseases. Biomed Res Int (2014) 2014:284836. 10.1155/2014/284836 24696846 PMC3947664

[68] Seatter SC Li MH Bubrick MP West MA. Endotoxin pretreatment of human monocytes alters subsequent endotoxin-triggered release of inflammatory mediators. Shock (1995) 3:252–8. 10.1097/00024382-199504000-00002 7600192

[69] Williams MA Withington S Newland AC Kelsey SM. Monocyte anergy in septic shock is associated with a predilection to apoptosis and is reversed by granulocyte-macrophage colony-stimulating factor *ex vivo*. J Infect Dis (1998) 178:1421–33. 10.1086/314447 9780264

[70] Waller T Kesper L Hirschfeld J Dommisch H Kolpin J Oldenburg J *Porphyromonas gingivalis* outer membrane vesicles induce selective tumor necrosis factor tolerance in a toll-like receptor 4 and mTOR-dependent manner. Infect Immun (2016) 84:1194–204. 10.1128/IAI.01390-15 26857578 PMC4807478

[71] Lucas H Bartold PM Dharmapatni AA Holding CA Haynes DR. Inhibition of apoptosis in periodontitis. J Dent Res (2010) 89:29–33. 10.1177/0022034509350708 19948942

[72] Shiratsuchi H Basson MD. Extracellular pressure stimulates macrophage phagocytosis by inhibiting a pathway involving FAK and ERK. Am J Physiol Cell Physiol (2004) 286:C1358–66. 10.1152/ajpcell.00553.2003 14761895

[73] Pike RN Potempa J McGraw W Coetzer TH Travis J. Characterization of the binding activities of proteinase-adhesin complexes from *Porphyromonas gingivalis*. J Bacteriol (1996) 178:2876–82. 10.1128/jb.178.10.2876-2882.1996 8631676 PMC178023

[74] Ito R Ishihara K Shoji M Nakayama K Okuda K. Hemagglutinin/Adhesin domains of *Porphyromonas gingivalis* play key roles in coaggregation with *Treponema denticola*. FEMS Immunol Med Microbiol (2010) 60:251–60. 10.1111/j.1574-695X.2010.00737.x 21039921

[75] Giacona MB Papapanou PN Lamster IB Rong LL D’Agati VD Schmidt AM *Porphyromonas gingivalis* induces its uptake by human macrophages and promotes foam cell formation in vitro. FEMS Microbiol Lett (2004) 241:95–101. 10.1016/j.femsle.2004.10.009 15556715

[76] Hajishengallis G Wang M Harokopakis E Triantafilou M Triantafilou K. *Porphyromonas gingivalis* fimbriae proactively modulate beta2 integrin adhesive activity and promote binding to and internalization by macrophages. Infect Immun (2006) 74:5658–66. 10.1128/IAI.00784-06 16988241 PMC1594907

[77] Polak D Shapira L Weiss EI Houri-Haddad Y. Virulence mechanism of bacteria in mixed infection: attenuation of cytokine levels and evasion of polymorphonuclear leukocyte phagocytosis. J Periodontol (2013) 84:1463–8. 10.1902/jop.2012.120528 23259412

[78] Hajishengallis G Liang S Payne MA Hashim A Jotwani R Eskan MA Low-abundance biofilm species orchestrates inflammatory periodontal disease through the commensal microbiota and complement. Cell Host Microbe (2011) 10:497–506. 10.1016/j.chom.2011.10.006 22036469 PMC3221781

[79] Orth RK O’Brien-Simpson NM Dashper SG Reynolds EC. Synergistic virulence of *Porphyromonas gingivalis* and *Treponema denticola* in a murine periodontitis model. Mol Oral Microbiol (2011) 26:229–40. 10.1111/j.2041-1014.2011.00612.x 21729244

[80] Hajishengallis G Lamont RJ. Beyond the red complex and into more complexity: the polymicrobial synergy and dysbiosis (PSD) model of periodontal disease etiology. Mol Oral Microbiol (2012) 27:409–19. 10.1111/j.2041-1014.2012.00663.x 23134607 PMC3653317

[81] Zhu Y Dashper SG Chen YY Crawford S Slakeski N Reynolds EC *Porphyromonas gingivalis* and *Treponema denticola* synergistic polymicrobial biofilm development. PLoS One (2013) 8:e71727 10.1371/journal.pone.0071727 23990979 PMC3753311

[82] Tan KH Seers CA Dashper SG Mitchell HL Pyke JS Meuric V *Porphyromonas gingivalis* and *Treponema denticola* exhibit metabolic symbioses. PLoS Pathog (2014) 10:e1003955. 10.1371/journal.ppat.1003955 24603978 PMC3946380

